# Comparing C2=O and C2=S Barbiturates: Different Hydrogen-Bonding Patterns of Thiobarbiturates in Solution and the Solid State

**DOI:** 10.3390/ijms222312679

**Published:** 2021-11-24

**Authors:** Chenming Li, Philipp Hilgeroth, Nazmul Hasan, Dieter Ströhl, Jörg Kressler, Wolfgang H. Binder

**Affiliations:** 1 Macromolecular Chemistry, Institute of Chemistry, Martin-Luther University Halle-Wittenberg, Von-Danckelmann-Platz 4, D-06120 Halle (Saale), Germany; chenming.li@chemie.uni-halle.de (C.L.); philipp.hilgeroth@chemie.uni-halle.de (P.H.); 2 Physical Chemistry, Institute of Chemistry, Martin-Luther University Halle-Wittenberg, Von-Danckelmann-Platz 4, D-06120 Halle (Saale), Germany; nazmul.hasan@chemie.uni-halle.de (N.H.); joerg.kressler@chemie.uni-halle.de (J.K.); 3 Organic Chemistry, Institute of Chemistry, Martin-Luther University Halle-Wittenberg, Kurt-Mothes-Str. 2, 06120 Halle (Saale), Germany; dieter.stroehl@chemie.uni-halle.de

**Keywords:** (2-thio)barbiturates, hydrogen bonds, supramolecular association, polyisobutylene, Langmuir film

## Abstract

Carbonyl-centered hydrogen bonds with various strength and geometries are often exploited in materials to embed dynamic and adaptive properties, with the use of thiocarbonyl groups as hydrogen-bonding acceptors remaining only scarcely investigated. We herein report a comparative study of C2=O and C2=S barbiturates in view of their differing hydrogen bonds, using the 5,5-disubstituted barbiturate **B** and the thiobarbiturate **TB** as model compounds. Owing to the different hydrogen-bonding strength and geometries of C2=O vs. C2=S, we postulate the formation of different hydrogen-bonding patterns in C2=S in comparison to the C2=O in conventional barbiturates. To study differences in their association in solution, we conducted concentration- and temperature-dependent NMR experiments to compare their association constants, Gibbs free energy of association *∆G_assn._*, and the coalescence behavior of the N-H‧‧‧S=C bonded assemblies. In Langmuir films, the introduction of C2=S suppressed 2D crystallization when comparing **B** and **TB** using Brewster angle microscopy, also revealing a significant deviation in morphology. When embedded into a hydrophobic polymer such as polyisobutylene, a largely different rheological behavior was observed for the barbiturate-bearing **PB** compared to the thiobarbiturate-bearing **PTB** polymers, indicative of a stronger hydrogen bonding in the thioanalogue **PTB**. We therefore prove that H-bonds, when affixed to a polymer, here the thiobarbiturate moieties in **PTB,** can reinforce the nonpolar PIB matrix even better, thus indicating the formation of stronger H-bonds among the thiobarbiturates in polymers in contrast to the effects observed in solution.

## 1. Introduction

Hydrogen bonds (H-bonds) are non-covalent attractive interactions between hydrogen atoms and electronegative elements/groups [1] and are very prominent in supramolecular science to achieve molecular ordering effects and recognition. The formation of simple H-bonds is described as a proton-sharing process (D-H‧‧‧:A↔D:^−^ ‧‧‧H-A^+^) between a Lewis acid (donor) and a Lewis base (acceptor) [2], where often oxygen or nitrogen act as a H-bond acceptor element. However, also other main group elements like sulfur [3,4,5,6,7] and selenium [7,8] were reported as potential acceptors despite their comparatively lower electronegativity. The use of thiocarbonyl groups (C=S) as hydrogen-bonding (H-bonding) acceptors instead of the carbonyl group (C=O) displays significantly different assembly behavior, as they form tilted H-bonding patterns, subsequently useful for organocatalysis [9]. The use of thioamides to replace natural amides as a weaker H-bonding acceptor can change the folding mechanisms in proteins [10], and different oblique H-bonding layers can form as Langmuir films when using thioureas instead of ureas [11]. In the area of materials displaying adaptive [12,13,14] and dynamic properties [15,16], the use of H-bonds bearing thiocarbonyl moieties has also become attractive due to their unique assembly pattern. Thus the replacement of urea groups by thiourea groups generates non-linear H-bonds, which promote dynamic crosslinking, reduce crystallization, and thus can impart improved self-healing properties to materials [15]. Several biomimetic polymer networks were therefore designed based on thiourea bonds, which exhibit solid-state plasticity and reprocessability in addition to the formation of stiff, strong, tough, and resilient mechanical properties [16].

When looking towards multiple carbonyl groups, barbiturates display a well-known H-bonding entity featuring an ADA/DA motive (D = donor; A = acceptor). They can bind specifically and strongly towards artificial receptors like the Hamilton Wedge [17,18,19], triazines [20], and diaminopyridines [21], display self-association [22,23,24], and generate transient H-bonds networks in barbiturate-bearing polymers [25,26], despite their comparatively low association constant in nonpolar solvents of only 23.6 M^−1^ (allobarbital) [22]. When attaching them onto (macro-)molecules, unique dynamic properties via their distinctive H-bonding in functional materials can be achieved, such as in monolayers [27,28,29], in coordination complexes [30,31], in 3D printing [32], or as self-healing-polymers [25,26,32,33,34] in self-assembled materials [35,36].

**Scheme 1:**
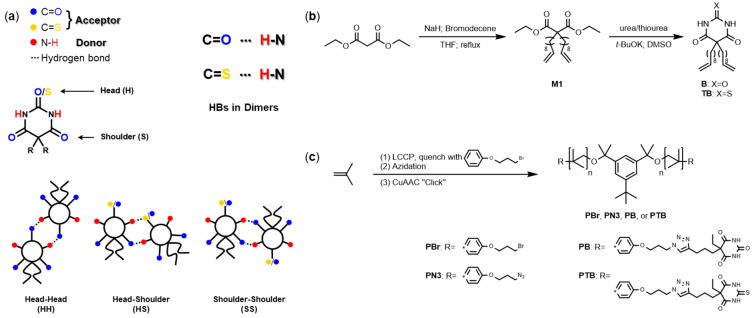
(**a**) The possible association of (thio-)barbiturates via H-bonding between N-H and C=O/S; (**b**) synthesis of the model compounds **B** and **TB**; and (**c**) the polyisobutylene (PIB)-based model polymers **PB** and **PTB**. THF: tetrahydrofuran; *t*-BuOK: potassium tert-butoxide; DMSO: dimethyl sulfoxide; LCCP: living carbocationic polymerization; CuAAC: copper(I)-catalyzed alkyne-azide cycloaddition ‘‘click’’ reaction.

While the H-bonds in native barbiturates were systematically studied widely before, the molecular exchange of thiocarbonyl moieties (C=S) in barbiturates to understand H-bonds has not been intensely exploited yet [37,38,39]. This motivated us to investigate thiobarbiturates and their H-bonding behavior in more detail. We herein report a comparative study of C2=O and C2=S barbiturates in view of their divergent H-bonds, using the 5,5-disubstituted barbiturate **B** and the thiobarbiturate **TB** as model compounds. We expected the formation of different H-bonding patterns due to the fixation of the C=S group in comparison to the C=O group in normal barbiturates as shown in Scheme 1. To study differences in their association in solution and the solid state, we conducted investigations on the association constants of **B** and **TB** via concentration-dependent NMR experiments to compare their Gibbs free energy of association $\Delta G_{assn.}$. Furthermore, we generated Langmuir films as a 2D material, where both **B** and **TB** displayed a different aggregation behavior observed by Brewster angle microscopy (BAM). To understand the H-bonds’ behavior in the melt state, we also affixed the (thio-)barbiturate moieties onto bulk polyisobutylene (named as **PB** for barbiturate-bearing polyisobutylene and **PTB** for thiobarbiturate-bearing polyisobutylene), revealing a largely different rheological behavior, again indicative for the distinct H-bonding of the C=S-moieties in comparison to the C=O-barbiturates.

## 2. Results

### Synthesis

We firstly prepared the model compounds **B** and **TB**, differing only in the C2 position, either containing a C=O or a C=S group. The syntheses (for details, see Supplementary Materials) were accomplished using diethyl malonate and 10-bromo-1-decene as starting materials, followed by condensation with an excess amount of either urea or thiourea in DMSO. For the model polymers **PB** and **PTB**, the diethyl ethylmalonate was charged with 5-chloro-1-pentyne to synthesize the (thio-)barbiturates for CuAAc “click” reaction with a telechelic diazidopolyisobutylene, thus affixing two thiobarbiturate moieties to either end of the polymer chain. The structure of the two model systems **B** and **TB** and **PB** and **PTB** were unambiguously proven by NMR-, IR spectroscopy, and ESI- or MALDI-ToF mass spectroscopy (for spectra, see Figures S3–S11), thus allowing for further investigations of H-bonding, which are discussed in the next chapters.

## 3. Discussion

### 3.1. Hydrogen Bonding of Model Compounds in Solution

As a first step to understand the behavior of the H-bonds in solution, we studied the low molecular weight systems via the model compounds **B** and **TB** to understand basic differences in strength and dynamics of association in non-competing chloroform.

#### 3.1.1. Concentration-Dependent Assembly Studies in Solution

**Figure 1:**
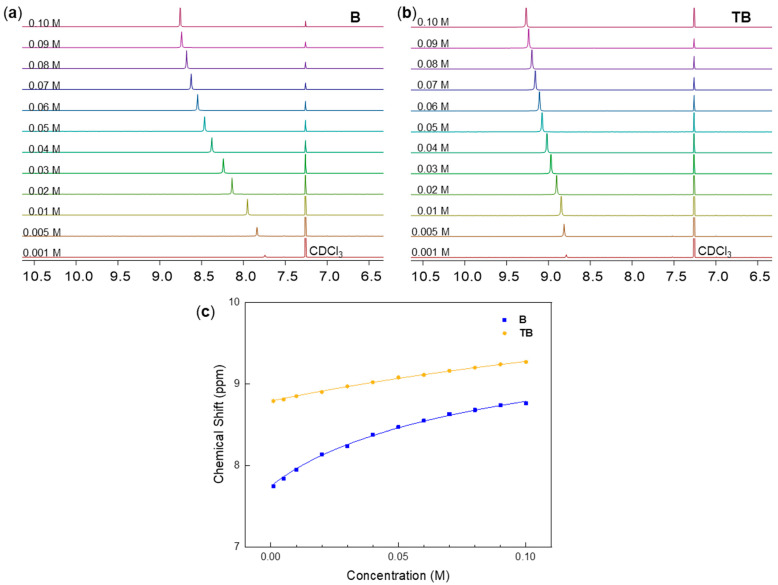
(**a**,**b**) Concentration-dependent NMR spectra of model compounds **B** and **TB** in CDCl_3_ measured at 27 °C and (**c**) their fitting curves of the chemical shifts of the N-H (for fitting details, see Supplementary Materials).

Barbiturates can form dimeric assemblies in inert solvents like chloroform [17,25] with a *K_assn._* < 100 M^−1^ or even lower. Using NMR spectroscopy under diluting conditions the association constant can be determined and compared to reported values of the self-association of barbitals [22]. For both model compounds **B** and **TB** the chemical shifts of their NH protons were observed by NMR spectroscopy, in turn determining their association constants (dimerization) by nonlinear fitting of the chemical shifts, as shown in Figure 1 (for fitting details, see Supplementary Materials).

From these nonlinear fitting of the N-H proton chemical shifts, the association constants (see Table 1) were determined as 4.26 M^−1^ (for **B**) and 0.94 M^−1^ (for **TB**), counting to a nearly 4-fold decrease by the introduction of the C=S group. This clearly indicates a weaker H-bonding with the C=S group, in line with our expectations. Looking at the donor-capabilities of the N-H in **TB**, it could be regarded as more acidic than in **B** (*pK*_HA,Urea_ = 26.9 and *pK*_HA,Thiourea_ = 21.0 [40]), thus placing **TB** as a better H-bond donor as, e.g., reported for thioamides [41]. Herein, self-consistent reaction-field calculations (SCRF) showed that a (C=S)N-H‧‧‧O=C-(NH) interaction in thioacetamides is 3.3–4.6 kcal‧mol^−1^ more stable than the (C=O)N-H‧‧‧S=C-(NH) orientation, but also more stable (by 1.5 kcal‧mol^−1^) when compared to the (C=O)N-H‧‧‧O=C-(NH) H-bond (present in conventional acetamide) [41]. Although the π-system in thiocarbonyls is regarded an effective acceptor and could form out-of-plan H-bonds [5].Thio-compounds generally bear a lower H-bonding basicity than their oxo-analogues [42,43], resulting in a weaker acceptor nature of the thio-species. Based on the observed 4-fold decrease in the association constant of **TB,** the latter thus seems to surpass the former argument about the H-bonding in **TB**. When comparing the tendency towards the hydrogen-bonded (H-bonded) assemblies, the negative Gibbs free energy of association Δ*G_assn._* of **B** evidences stronger H-bonds, whereas nearly equilibrated associated/free N-H in **TB** at 27 °C in chloroform can be observed.

#### 3.1.2. Temperature-Dependent Association Behavior

**Figure 2:**
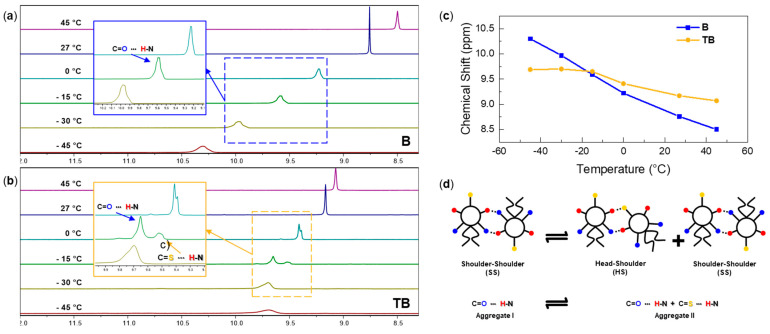
(**a**,**b**) Temperature-dependent ^1^H NMR spectra of model compounds **B** and **TB** in CDCl_3_, (**c**) chemical shifts of N-H vs. temperature of the model compounds **B** and **TB**, and (**d**) the possible exchangeable assemblies in thiobarbiturate **TB** in CDCl_3_ at *T_Coalescence_* = 0 °C.

As demonstrated in Scheme 1 and Figure 2d, (thio-)barbiturates can form several dimeric H-bonding patterns via the N1/3-H with the C2=O/S and C4/6=O carbonyls, forming dimers via ‘‘head–head’’ (HH), ‘‘head–shoulder’’ (HS), and ‘‘shoulder–shoulder’’ (SS) orientations. While the purely C=O-based barbiturates could form all three possible arrangements, dimers of the thiobarbiturates could have preferred orientations due to the lower H-bonding acceptor qualities of the thiocarbonyl group [42,43] and the different lone-pair electron density at the sulfur atom [44]. Due to the dynamic nature of aggregates, the H-bonding arrangements are continuously exchanging, expecting a significant change in dynamics at reduced temperatures. To detect the dynamic H-bonding of the N-H‧‧‧O/S=C in the dimer of the model compounds **B** and **TB**, temperature-dependent NMR experiments were conducted to evaluate an eventual coalescence behavior of the dynamic assemblies.

In Figure 2a,b, the temperature-dependent ^1^H NMR spectra of both model compounds **B** and **TB** are shown. Starting from 0°C for the thiobarbiturate **TB**, we clearly observe a splitting of the NH protons, indicative for two differently ordered arrangements—a behavior that was not observed for the native barbiturate **B**. It can be hypothesized that this is an indication for the formation of the two types of aggregates, I and II (see Figure 2d), which contain SS orientation or both HS and SS orientations, respectively. A coalescence temperature of *T_Coalescence_* = 0 °C is observed, above which the two arrangements HS and SS would interchange faster compared to the NMR timescale and, thus, we found coalesce with a rate constant *k_C_* = 205.1 s^−1^ (for calculation details, see Supplementary Materials).

By calculating the ratio between the integral of the two isolated N-H peaks, the exchange equilibrium constant *K*_−15°C_ of the aggregates I and II at −15 °C could be obtained (see Table 1). With *K*_−15°C_ = 0.32 M^−1^, we concluded that, at a temperature lower than *T_Coalescence_*, **TB** tends to form dimers with a predominant N-H‧‧‧O=C instead of the N-H‧‧‧S=C orientation. At temperatures around −30 °C, the peak representing the N-H‧‧‧S=C bond, believed to be the weaker H-bonds and in an even slower exchange regime, is no longer detectable by NMR spectroscopy, presumably by peak broadening and overlap. There is no significant change of the N-H chemical shift of **TB** at temperatures lower than −15 °C (see Figure 2c), indicative that the equilibrium is frozen in **TB,** while the still decreasing N-H chemical shift of **B** demonstrates the dynamic nature of the H-bonded aggregates in **B** in this temperature range.

Overall, we see an indication for the participation of the C=S thiocarbonyl groups in the dimerization of **TB**, in total resulting in a 4-fold decrease in its overall dimerization in chloroform. While the timescale of the exchangeable aggregates in **B** is too fast to be monitored by NMR spectroscopy, the coalescence behavior of **TB** aggregates is observed, indicating aggregates in **TB** with a preferred ‘‘SS’’ orientation at temperatures below *T_Coalescence_*. Thus, by replacing C2=O with C2=S, there is a change in the molecular arrangements and the overall strength of the H-bonds.

### 3.2. Hydrogen Bonding of Model Compounds in the Solid State

As the strength and arrangement of H-bonds differs significantly when moving to the solid state, we also studied the behavior of **B** vs. **TB** in the solid state. Here, the H-bonds in the bulk model compounds **B** and **TB** were first investigated via temperature-dependent ATR FT-IR experiments, and subsequently as Langmuir monolayers in a 2D arrangement.

#### 3.2.1. Hydrogen Bonds in Model Compounds B and TB at Elevated Temperatures

**Figure 3:**
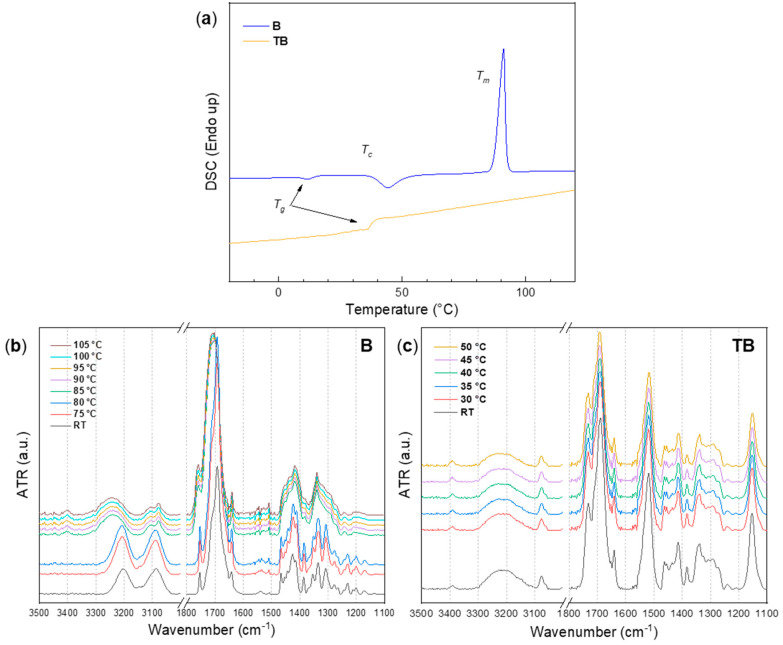
(**a**) DSC curves (heating rate of 5 K/min, the thermal history was cancelled by the preheating and cooling circle); (**b**),**c**) the ATR FT-IR spectra at various temperatures of model compounds **B** and **TB**. *T_g_*: glass transition temperature; *T_c_*: crystallization temperature; *T_m_*: melting temperature.

To understand the potential contribution of the C=S moiety in these H-bonding arrangements, we used temperature-dependent FT-IR spectroscopy for clarification. Due to the presence of the two 10-carbon hydrocarbon chains at the C5 position in both model compounds, **B** and **TB** show a glass transition (*T_g_*) (for **B** and **TB**) (see Figure 3a), with only **B** displaying crystallization (*T_c_*) and melting (*T_m_*). To study the H-bonds in the solid state at elevated temperatures, temperatures around the melting peak of **B** and the glass transition of **TB** were selected to see how the H-bonds vary below and above the respective *T_g_*, where molecular motions are either frozen or enabled. Below the melting in **B,** the free N-H stretching at ~3400 cm^−1^ is absent, whereas it is present in **TB** even at temperatures below its *T_g_*, which is an indication for the weaker association of the thiobarbiturates. While for **B,** the H-bonded N-H stretching shows a dual peak at 3200 and 3100 cm^−1^ [45], denoting the ordered H-bonds in barbiturates, in **TB,** the H-bonded N-H shows a much broader peak centered at ~3200 cm^−1^ with weak N-H symmetric stretching at ~3075 cm^−1^, indicating a more complex H-bonding pattern due to the existence of the C2=S [46,47,48] in the thiobarbiturates. The red-shifted resonance of the C=S band clearly proves that the C2=S is indeed H-bonded with the N-H [24,47,48,49]. As temperature rises above 85 °C, **B** melts partially, showing the free N-H stretching at ~3400 cm^−1^, together with a blueshift of the carbonyl stretching from 1692 to 1706 cm^−1^, demonstrating that the H-bonds in **B** are partially broken at elevated temperatures. As for **TB**, the IR spectra remain almost identical to those at room temperature, similar to the spectra of **B** in the melt state, indicative for an already dynamic character with more broken H-bonds in thiobarbiturates **TB** within the whole temperature range.

Owing to the strong and ordered H-bonds in barbiturates, barbiturate **B** presents as a white powder that can form crystals, which is consistent with previous reports to use the H-bonds to engineer its ordering via crystallization [15,50,51]. Thiobarbiturate **TB**, in contrast, presents itself as a highly viscous liquid at room temperature. This difference again gives evidence for the significantly different dynamic nature of C2=O vs. C2=S in barbiturates, as the H-bonds change from an ordered crystalline nature to a rather amorphous behavior when introduced into solid materials.

#### 3.2.2. Hydrogen Bonds in 2D-Ordered Films

**Figure 4:**
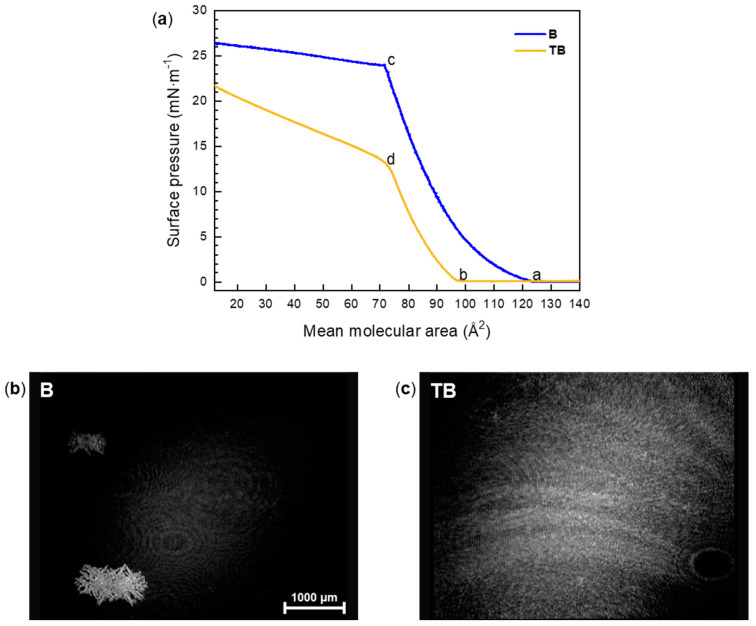
(**a**) *π-mmA* isotherm recorded on the water subphase at 20 °C with the compression rate of 1.5 Å^2^ / (molecule‧min); Table 124 Å^2^, b = ~ 97 Å^2^, c= ~ 71 Å^2^, and d = ~ 73 Å^2^. (**b**,**c**) BAM images of the Langmuir films at 15 Å^2^ of model compounds **B** and **TB**.

It was reported [27,28,29] that barbiturates can form Langmuir films on a water subphase; however, there is no report regarding their thio-analogues. Thus, a Langmuir film of either **B** and **TB** was generated by spreading the compound solutions on the water subphase, evaporating the solvent, and compressing the films by moving the barriers of the Langmuir trough. The surface pressure-mean molecular area (*π-mmA*) isotherms of the model compounds **B** and **TB** are shown in Figure 4a.

When compression starts from 140 Å^2^, both **B** and **TB** do not display an increase in surface pressure, indicating a gas-like state of the molecules on the water subphase. When the area reduces to ~124 Å^2^, the surface pressure of **B** starts to rise, which by coincidence roughly matches the theoretically calculated mean molecular area of 120 Å^2^ for a vertically lying head group on the water surface, with the hydrocarbon chains oriented into the air (for a space filling model, see Figure S2). For **TB**, the increase takes place at ~97 Å^2^, explained by the formation of molecular stacks of the film when compressed. After an increase with an almost identical slope, the transition occurs at 71 Å^2^ and 73 Å^2^ for **B** and **TB**, respectively, which could either be explained by crystallization or multilayer formation, since it was reported that the nature of the H-bonds can change during crystallization [15,50,51]. As shown in Figure 4b, several crystalline domains can clearly be observed for **B**, but most parts of the film remain amorphous. An explanation could be that the crystallization is the result from compression and the amorphous part of **B** is not visible due to the poor contrast against the water subphase, which is the limitation of BAM. Meanwhile for **TB**, clearly there is no crystallization and only ribbon-like structures are observed. Thus, we think that the strong H-bonds within the polar head group barbiturates in **B** indeed contribute to crystallization, which is also in line with the investigations reported above, where **B** shows a higher tendency to crystallize than **TB** owing to the stronger and more ordered H-bonds. Thus, by introducing the C2=S, the H-bonds are weakened and lead to the formation of amorphous aggregates even under compression. The observation of distinct domains in BAM motivates further, more detailed investigations to be reported in future work.

### 3.3. Hydrogen Bonding of Model Polymers

Based on our previous work on barbiturate-modified polyisobutylene [25,26,34,52,53], we were curious how the use of C2=S-modified barbiturates would change the materials properties. Especially the formation of different H-bonding patterns with the now known weaker association was expected to change their assembly behavior significantly. We therefore prepared the C=S-modified model polymer analogues similar to those prepared previously and studied their association behavior by melt rheology, where the C=O-containing barbiturates form aggregates as reported previously [25,26,52,53]. Thus, we prepared the polymers **PB** and **PTB**, both featuring a molecular weight Mn = ~3.2 kDa, bearing two identical (thio-)barbiturates at either of the chain ends. The synthesis was based on the strategy via living carbocationic polymerization, followed by end group modification and subsequent CuAAc “click” reaction to attach the (thio-)barbiturate moieties [25] (for synthesis, see Figure S2). Purity and properties of the so-modified model polymers **PB** and **PTB** were proven by NMR, GPC, and MALDI-ToF mass spectroscopy (for spectra and characterization data, see Figures S5,S6,S10 and S11, and Table S1).

**Figure 5:**
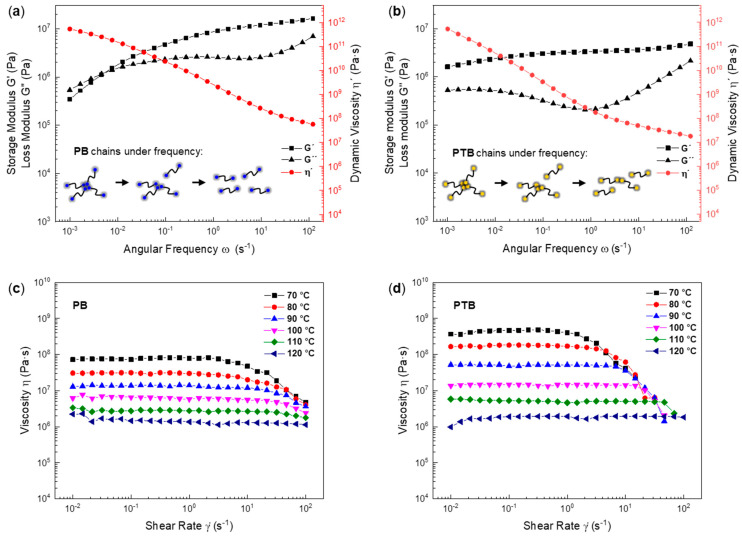
Storage modulus *G′*, loss modulus *G″*, and dynamic viscosity *η*′ vs. angular frequency *ω* measured at 20 °C, and viscosity *η* vs. shear rate $\overset{\;\;.}{\gamma }$ at elevated temperatures of the model polymer **PB** (**a**,**c**) and **PTB** (**b**,**d**).

To understand differences of the H-bonding in the polymer matrix, the polymers modified with barbiturates (**PB**) and 2-thiobarbiturates (**PTB**) were subjected to melt rheology. As known from the model compounds in solution, we sought to analyze their clustering behavior as shown in Figure 5. At room temperature, while the precursor polymer **PBr** (telechelic dibromo PIB) is a viscous liquid, both the model polymer **PB** and **PTB** present themselves as rubbery materials due to the clustering of the end groups induced by the H-bonds. In both polymers, the glass transition temperature was only slightly increased from −58.8 °C (for **PBr**) to −61.3 °C and −59.6 °C for **PB** and **PTB**, respectively (for DSC curves, see Figure S12). The rubbery nature originates from the association of the barbiturate/thiobarbiturate end groups via their H-bonds acting as “stickers”, which connect the soft polyisobutylene chains into transient H-bonding networks. The lifetime *τ* of the ‘‘stickers’’, namely the lifetime of the aggregates bonded via their H-bonds, can be characterized by the crossover point in frequency sweep rheological measurement [54]. Interestingly, as shown in Figure 5a, **PB** shows terminal flow at 20 °C with a crossover point at *τ* = 5.27 × 10^−3^ s^−1^, with the dynamic viscosity *η′* being increased to ~10^7^ Pa‧s. However, for **PTB**, terminal flow is not observed at 20 °C. Due to the absence of the crossover within the given frequency range, one can conclude that the thiobarbiturate aggregates in **PTB** display a longer lifetime compared to that of the native barbiturate aggregates in **PB**. Besides their longer lifetime, H-bonds in thiobarbiturates are indeed more tolerant against a low frequency (of shear) than those in barbiturates at frequency lower than ~53 s^−1^, evidenced by a lower loss tangent value of **PTB** than that of **PB** (for the loss tangent vs. frequency curve, see Figure S13). As represented in Figure 5a,b, during the frequency sweep, the **PB** chains, connected via the H-bonding network, are gradually opened by the shear-stress, causing an overall decrease in viscosity. The H-bonding network in **PTB**, to some extent, was also dynamized, but within the given frequency range the H-bonds network can still persist in the “more closed” state, indicating that at a certain frequency range the H-bonds in **TB** may be even as strong (or even stronger) inside the polymer when compared to the native barbiturate moieties.

Due to the rubbery nature of the model polymers at room temperature, the viscosities were determined from 70 °C to 120 °C and the viscosity vs. shear rate at elevated temperatures is shown in Figure 5c,d. Both polymers show a shear-thinning behavior, which is in line with the behavior observed in the melt extrusion of these polymers, as required for 3D printing [32]. At a temperature between 70 °C and 110 °C, **PTB** always demonstrates a higher zero-shear viscosity (for the zero-shear viscosity vs. temperature curve, see Figure S14) than that of **PB**, which could be another proof of the in view of stronger rheology H-bonds in thiobarbiturates than those in barbiturates, when applied in the nonpolar polyisobutylene matrix. The shear-thinning behavior disappears after the temperature reaches 120 °C, indicative for a complete rupture of the H-bonds within the network. Therefore, if free from H-bonding, the (thio-)barbiturate-modified **PB** and **PTB** show an almost identical native viscosity, and the unexpected higher viscosity in **PTB** may be a result of the weaker and more discrete H-bonds in thiobarbiturates instead of the stronger and more ordered H-bonds, which deviates from the observation in model compound solutions.

## 4. Materials and Methods

Urea was purchased from Merck; thiourea was purchased from Apolda (DDR); diethyl ethylmalonate was purchased from J&K (Germany); sodium azide, sodium hydride, copper(I) iodide, and potassium tert-butoxide were purchased from Sigma Aldrich (Germany); diethyl malonate, 10-bromo-1-decene, 5-cloro-1-pentyne, and diisopropylethylamine were purchased from TCI (Belgium). All chemicals listed here were used without any purification unless otherwise stated. Solvents handling and the chemicals that are not mentioned here can be found in the Supplementary Materials.

### 4.1. Nuclear Magnetic Resonance Spectroscopy (NMR)

All ^1^H-NMR and ^13^C-NMR spectra were measured on a Varian FT-NMR spectrometer (500 and 101 MHz, respectively), Agilent Technologies (Agilent Technologies Germany GmbH & Co. KG, Waldbronn, Germany). All samples were measured at 27 °C using deuterated chloroform (CDCl_3_) or DMSO-d_6_. Chemical shifts (δ) were recorded in parts per million (ppm) relative to the remaining solvent signals (CDCl_3_: 7.26 ppm (^1^H) and 77.0 ppm (^13^C), DMSO-d_6_: 2.50 ppm (^1^H) and 39.5 ppm (^13^C)). Chemical shifts were reported with the following notations: s, singlet; d, doublet; t, triplet; q, quartlet; p, pentlet; m, a more complex multiplet or overlapping multiplets. The data analysis was performed on the software MestReNova (version 9.0.1-13254).

### 4.2. Differential Scanning Calorimetry (DSC)

DSC measurements were accomplished on a Netzsch DSC 204 F1 Phoenix (NETZSCH-Gerätebau GmbH, Selb, Germany). Samples pieces with a mass of 5–10 mg were placed into aluminum crucibles and were heated under nitrogen atmosphere. The thermal history was cancelled by a preheating circle to 150 °C with a rate of 10 K/min, then keeping the sample for 20 min at 150 °C, followed by cooling to −50 °C with a rate of 5 k/min, then keeping the sample for 20 min at −50 °C. The DSC data were collected from the second heating circle from −50 °C to 150 °C with a heating rate of 5 K/min. Data analysis was performed on the software NETZSCH Proteus (version 5.2.1.) and Origin 2018 (version b9.5.0.193).

### 4.3. ATR FT-IR Spectroscopy

ATR-FTIR spectra were recorded using a Bruker Tensor vertex 70 (Bruker Optik GmbH, Bremen, Germany) equipped with a Golden Gate Heated Diamond ATR top plate (Specac Ltd, Orpington, UK). All wavenumbers are given in cm^−1^. For FT-IR measurements at elevated temperatures, samples were heated with the heating rate of 10 K/min, and equilibrated at the desired temperature for 10 min before the measurement started. Data analysis was performed via the software OPUS (version 8.2) and Origin 2018 (version b9.5.0.193).

### 4.4. Langmuir Isotherm

The π-*mmA* isotherms were recorded using a Langmuir trough (Riegler & Kirstein GmbH, Potsdam, Germany) with a maximum trough area of 545 cm^2^. The trough was equipped with two moveable barriers and a Wilhelmy plate made of filter paper. The entire trough was covered by a Plexiglas box to maintain an equilibrium environment. Millipore water was used as a subphase for the experiment. The temperature of the subphase was kept at 20 °C using a thermostat. Before spreading the compound solutions, the purity of the subphase was checked by surface pressure measurement at a maximum barrier compression (π < 0.15 mN/m). Compound solutions with a concentration of ~1 mg/mL were prepared in chloroform and spread dropwise in some random locations on the subphase using a Hamilton digital syringe. After a 20 min waiting time for complete solvent evaporation, the trough surface was compressed at a speed of 1.5 Å^2^/(molecule‧min) to record the pressure–mean molecular area isotherms. Data analysis was performed via the software Origin 2018 (version b9.5.0.193).

### 4.5. Brewster Angle Microscopy

To monitor the water surface during compression, a Brewster angle microscope (NFT Mini BAM, Nanofilm Technologies, Valley View, OH, USA) coupled with a Langmuir trough of 142 cm^2^ was used. The lateral resolution of the microscopy was 20 µm with a field view of 4.8 × 6.4 mm^2^. The images were captured using the software WinTV (Hauppauge Inc, Hauppauge, NY, USA). The imaging of the Langmuir film was done at different surface pressures during the film compression at a rate of 1.5 Å^2^/(molecule‧min).

### 4.6. Rheology

Rheology experiments were performed on an MCR 101-DSO (Anton Paar Germany GmbH, Ostfildern-Scharnhausen, Germany) using a parallel plate–plate geometry (plate diameter 8 mm). All polymers were dried under high vacuum at 80 °C for 48 h before the rheology measurement. The solid samples were vacuum hot pressed into films at 80 °C, and then cut into discs of 8 mm with a punching tool. The sample temperature was regulated by thermoelectric cooling/heating in a Peltier chamber under a dry nitrogen atmosphere. At each temperature the sample was equilibrated for 20 min before the measurement was started. All measurements were performed in the dynamic mode and repeated for twice to ensure the precise viscosity values. The frequency sweeps were done within the linear viscoelastic regime (LVE) with 5% deformation applied. Data analysis was performed via the software Pheo Compass^TM^ (version V1.30.1064) and Origin 2018 (version b9.5.0.193).

Methods which are not mentioned here can be found in the Supplementary Materials (Figures S1–S14, Table S1).

## 5. Conclusions

In summary, we investigated the association behavior of the model compounds 5,5-disubstituted barbiturate **B** and thiobarbiturate **TB** in the nonpolar solvent chloroform. By replacing the C2=O with the C2=S group, a 4-fold decrease of the dimerization constant was observed. The observed coalescence behavior of the H-bonded aggregates in **TB** with a *k_C_* = 205.1 s^−1^ at 0 °C via NMR spectroscopy clearly indicates the formation of at least two different orientations of the H-bonding patterns—an effect not observed in the conventional barbiturate **B**. In the bulk, the H-bonds of the model system were investigated by ATR FT-IR spectroscopy, together with DSC, demonstrating more ordered H-bonds in native barbiturate **B** and more discrete H-bonds in the thiobarbiturate **TB** at room temperature asindicated by the crystalline nature of **B** and the purely amorphous nature of **TB**. The Langmuir isotherms of the model compounds again demonstrate the tendency of the barbiturate **B** to crystallize due to the more ordered and stronger H-bonds, while the weaker H-bonds in C2=S of the thiobarbiturate **TB** suppress crystallization and facilitate the formation of molecular stacks, as evidenced by the surface pressure isotherm and the BAM images. When covalently bound to both ends of a linear, nonpolar polymer, the thiobarbiturate-modified **PTB** shows a longer lifetime under frequency sweep, in line with the observation from the model system in solution. **PTB** shows a higher zero-shear viscosity when compared to **PB**, diverging from the observation in small molecule model system where **B** possesses a higher association constant in comparison to **TB**. Thus, within the bulk- polymer thiobarbiturates offer an ‘‘as strong’’ or ‘‘even stronger’’ ‘‘sticker effect’’ compared to the native barbiturate-modified **PB**—a point often misjudged by the results obtained for small molecules in solution. When turning towards bulk systems, such deviation in bonding strength and orientation can be significant, showing the necessity for studies in the solid state rather than the solution state to understand the formation of dynamic and adaptive effects in materials. This work proves that, while thiobarbiturates demonstrate a weaker association in chloroform, the discrete H-bonds in thiobarbiturate-modified **PTB** can at least equally, or even better, reinforce the nonpolar polymer matrix, thus indicating the formation of stronger H-bonds of the thiobarbiturates in polymers in contrast to effects observed in solution. In line with the already reported additional formation of additional (weaker) out-of-plane H-bonds [5], this altogether can explain why the H-bonds in **PTB** are overall stronger than in **TB**, but are still highly dynamic as they could “slide-along” several binding partners, similar to the observation by Aida et. al. for their self-healing thiourea system [15]. It also underscores that the presence of both, amide-type and thioamide-type groups could be regarded as advantageous, as both dynamic features are present and thus control the dynamics of the material. Overall, this gives a hint to the often superior properties of thio-based H-bonds in some self-healing materials, opening a potential tool to engineer such materials with adaptive and responsive properties.

## Figures and Tables

**Table 1:** Association constant *K_assn._*, maximum chemical shift of N-H *δ_max_*, Gibbs free energy of association $\Delta G_{assn.}$, coalescence rate constant *k_C_*, Gibbs free energy of coalescence $\Delta G_{c}^{\neq }$, and exchange equilibrium constant *K*_−15°C_ of model compounds **B** and **TB**.

	*K_assn._*/M^−1^	*δ_max_*/ppm	Δ*G_assn._*/kJ‧mol^−1^	*k_C_*/s^−1^	$\Delta G_{c}^{\neq }/\mathrm{kJ}\cdot {\mathrm{mol}}^{-1}$	*K*_−15°C_/M^−1^
**B**	4.26 ± 0.40	10.67 ± 0.15	−3.61 ± 2.29	-	-	-
**TB**	0.94 ± 0.16	12.27 ± 0.46	0.15 ± 4.57	205.08	54.57	0.32

## Data Availability

Data are contained within the article.

## Supplementary Materials

The following are available online at https://www.mdpi.com/article/10.3390/ijms222312679/s1.
