# Supplementary material for: Comparing C2=O and C2=S Barbiturates: Different Hydrogen-Bonding Patterns of Thiobarbiturates in Solution and the Solid State
Source: Int J Mol Sci. 2021 Nov 24;22(23):12679. doi: 10.3390/ijms222312679 (PMC8657569; doi:10.3390/ijms222312679)
Supplement: Supplementary file 1 [file ijms-22-12679-s001.zip › ijms-1468860-supplementary.pdf]

Supporting Information

Comparing C2=O and C2=S Barbiturates: Different Hydrogen Bonding Patterns of Thiobarbiturates in Solution and the Solid State

Chenming Li[1], Phillip Hilgeroth[1], Nazmul Hasan[2], Dieter Ströhl[3], Jörg Kressler[2], and Wolfgang H. Binder[1]*

[1] Institute of Chemistry, Martin-Luther-University Halle-Wittenberg, Von-Danckelmann-Platz 4, D-06120 Halle (Saale), Germany; chenming.li@chemie.uni-halle.de
[2] Institute of Chemistry, Martin Luther University Halle-Wittenberg, Von-Danckelmann-Platz 4, D-06120 Halle (Saale), Germany; nazmul.hasan@chemie.uni-halle.de
[3] Institute of Chemistry, Martin Luther University Halle-Wittenberg, Kurt-Mothes-Str. 2, 06120 Halle (Saale), Germany; dieter.stroehl@chemie.uni-halle.de
* Correspondence: wolfgang.binder@chemie.uni-halle.de

1. EXPERIMENTAL PART ········ **1**

1.1. SOLVENTS AND MATERIALS ········ 1

1.2. METHODS ········ 1

1.3. SYNTHESIS ········ 3

*1) Synthesis of Diethyl 2,2-Di(dec-9-en-1-yl)malonate (**M1**)* ········ *3*

*2) Synthesis of Diethyl 2,2-Di(dec-9-en-1-yl)malonate (**M2**)* ········ 4

General Synthesis of Barbiturate and 2-Thiobarbiturate ········ 4

*3) Synthesis of 5,5-Di(dec-9-en-1-yl)pyrimidine-2,4,6(1H,3H,5H)-trione (**B**)* ········ 5

*4) Synthesis of 5,5-Di(dec-9-en-1-yl)-2-thioxodihydropyrimidine-4,6(1H,5H)-dione (**TB**)* ········ 5

*5) Synthesis of 5-Ethyl-5-(pent-4-yn-1-yl)pyrimidine-2,4,6(1H,3H,5H)-trione (**B2**)* ········ 5

*6) Synthesis of 5-Ethyl-5-(pent-4-yn-1-yl)-2-thioxodihydropyrimidine-4,6(1H,5H)-dione (**TB2**)* ········ *5*

Synthesis of Telechelic Polyisobutylenes ········ 6

*7) Synthesis of Dimethyl-5-(tert-butyl)isophthalate (**1**)* ········ 6

*8) Synthesis of 2,2-(5-(Tert-butyl)-1,3-phenylene)bis(propan-2-ol) (**2**)* ········ 6

*9) Synthesis of 1-(Tert-butyl)-3,5-bis(2-methoxypropan-2-yl)benzene (**3**)* ········ 7

*10) Synthesis of Telechelic Dibromopolyisobutylene (**PBr**)* ········ 7

*11) Synthesis of Telechelic Diazidopolyisobutylene (**PN3**)* ········ 8

*12) Synthesis of Telechelic Dibarbiturate Polyisobutylene (**PB**)* ········ 8

*13) Synthesis of Telechelic Dithiobarbiturate Polyisobutylene (**PTB**)* ········ 9

2. NONLINEAR FITTING OF NH CHEMICAL SHIFT FOR ASSOCIATION CONSTANTS ········ **10**

3. CALCULATION OF COALESCENCE CONSTANT ········ **10**

4. MOLECULE MODEL OF B IN LANGMUIR FILM ········ **11**

5. SUMMARY OF CHARACTERIZATION OF MODEL POLYMERS ········ **11**

6. NMR SPECTRA OF MODEL COMPOUNDS AND POLYMERS ········ **12**

7. ESI-TOF MS SPECTRA OF MODEL COMPOUNDS ········ **15**

8. MALDI-TOF MS SPECTRA OF MODEL POLYMERS ········ **16**

8.1. SPECTRA OF **PB** ········ 16

8.2. SPECTRA OF **PTB** ········ 17

9. DSC CURVES OF MODEL POLYMERS ········ **18**

10. LOSS TANGENT VS FREQUENCY CURVE OF MODEL POLYMERS ········ **18**

11. ZERO-SHEAR VISCOSITY VS TEMPERATURE CURVE OF MODEL POLYMERS · **19**

12. REFERENCE ········ **20**

1. Experimental Part

1.1. Solvents and Materials

Chloroform from VWR, ethyl acetate from Overlack, methanol from Brenntag, and toluene from Roth were purchased in technical grade and distilled at least once prior use. Deuterium chloroform ($CDCl_3$-*d*) and deuterium dimethyl sulfoxide (DMSO-*d6*), from Chemotrade, were used as NMR deuterium solvents.

Dry solvents were prepared as follows: tetrahydrofuran (THF), from Roth, was predried over potassium hydroxide for several days and refluxed over sodium and benzophenone under inert atmosphere and distilled freshly before use; *n*-hexane, from Roth, was refluxed over concentrated sulfuric acid and oleum to remove olefins and subsequently distilled over sodium and benzophenone under an inert gas atmosphere for several hours; dichloromethane (DCM), from Overlack, was predried over calcium chloride for several days and then refluxed over calcium hydride for several hours; diethyl ether, from Overlack, was passed through a column filled with sodium sulfate to remove moisture; dimethyl sulfoxide (DMSO), from Grüssing, was stored over molecular sieves (pore diameters 4Å) for several days before use; N,N-dimethylformamide (DMF) from Grüssing was stored over calcium hydride for several days before use.

1-Bromo-3-phenoxypropane was purchased from Alfa Aesar; titanium tetrachloride, magnesium chips, iodomethane, N,N-dimethylacetamide, 2,6-di-tert-butyl pyridine, and isobutylene were purchased from Sigma Aldrich; 5-(tert-butyl)isophthalic acid were purchased from TCI. All chemicals listed here were used without any purification unless otherwise stated.

1.2. Methods

Thin-layer Chromatography (TLC)

TLC was performed on "Merck silica gel 60" plates. Spots on TLC plate were visualized using UV light (254 or 366 nm), oxidizing agent "blue stain", or potassium

permanganate solution. "Blue stain" was prepared as follow: $(NH_4)_6Mo_7O_{24}\cdot 4H_2O$ (1 g) and $Ce(SO_4)_2\cdot 4H_2O$ (1 g) were dissolved in a mixture of distilled water (90 mL) and concentrated sulfuric acid (6 mL). Potassium permanganate solution was prepared as follow: $KMnO_4$(3 g) and K_2CO_3 (10 g) were dissolved in distilled water (300 ml).

Electrospray Ionization Time-of-Flight Mass Spectroscopy (ESI-ToF MS)

ESI-ToF MS measurements were performed using a Bruker Daltonics microTOF. 0.1 mg of samples were dissolved in HPLC grade methanol. All spectra were obtained by means of direct injection with a flow rate of 180 µL h-1 in the negative mode with an acceleration voltage of 4.5 kV.

Gel Permeation Chromatography

GPC measurements were performed at 40 °C on a Viscotek GPCmax VE 2002 from ViscotekTM applying a HHRH Guard-17369 and a GMHHR-N-18055 column. As solvent THF was used and the sample concentration was adjusted to 3 $mg\cdot mL^{-1}$ while applying a flow rate of 1 $mL\cdot min^{-1}$. For determination of the molecular weights the refractive index of the investigated sample was detected with a VE 3580 RI detector of ViscotekTM and PIB-standards (320 $g\cdot mol^{-1}$ to 578,000 $g\cdot mol^{-1}$) were used as reference from ViscotekTM.

Matrix-assisted Laser Desorption / Ionization Time-of-Flight Mass Spectrometry (MALDI-ToF MS)

MALDI-ToF MS was done on a Bruker Autoflex III system in the reflection mode. Formation of ions was obtained by laser desorption (smart beam laser at 355, 532, 808, and 1064 ± 6.5 nm; 3 ns pulse width; up to 2500 Hz repetition rate). Ions were accelerated by a voltage of 20 kV, and detected as positive ions. 1,8-dihydroxy-9,10-dihydroanthracen-9-one (Dithranol, 20 $mg\cdot mL^{-1}$ in THF) was used as matrix and sodium iodide (NaI, 20 $mg\cdot mL^{-1}$ in THF) was used as salts for ionizing polymers functionalized with hydrogen-bonding moieties (model polymer PB or PTB, 20 $mg\cdot mL^{-1}$ in THF) while applying a volume ratio of 100:20:1.

1.3. Synthesis

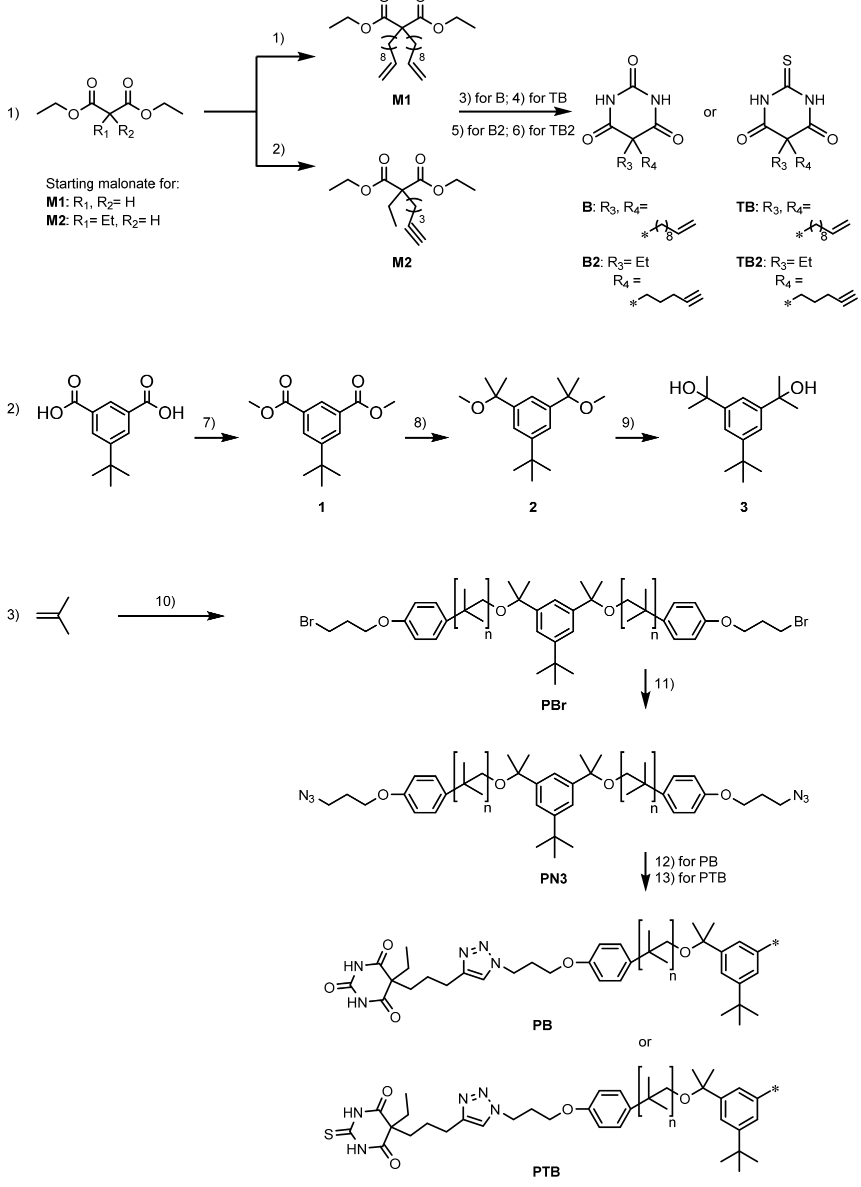

Figure S1 Synthetic procedure of 1) barbiturates **B**/**B2** and 2-thiobarbiturate **TB**/**TB2**; 2) the initiator **3** for LCCP of isobutylene; 3) telechelic dibarbiturate/dithiobarbiturate polyisobutylene **PB**/**PTB**.

1) Synthesis of Diethyl 2,2-Di(dec-9-en-1-yl)malonate (M1)

The synthesis of **M1** was modified according to reference [1]. To a two-necked flask, sodium hydride (0.88g, 60% wt dispersion in oil , 110 mmol) was placed followed by adding 20 ml dry THF. While the flask was cooled in an ice bath, diethyl malonate (1.52 ml, 10 mmol) dissolved in 5 ml dry THF was added dropwise. The reaction mixture was stirred for 30 minutes till clear, then treated with 10-bromo-1-decene (4.02 ml, 22 mmol) dissolved in 5 ml dry THF. The reaction mixture was heated to reflux

(temperature of oil bath was 85 °C) for 72 hours, monitored by TLC. After reaction was finished, the mixture was quenched with 50 ml 1 M hydrochloric acid, and was extracted with ethyl acetate (3 × 30 ml). The combined organic layers were washed with brine, dried with sodium sulfate, filtered and concentrated under reduced pressure. Column chromatography on silica with hexane/diethyl ether 100:10 was performed. After drying under vacuum, **M1** as a colorless liquid was obtained. Yield: 2.98 g (68 %). ^{1}H-NMR (500 MHz, $CDCl_3$): 5.78 (2 H, ddt, J 16.9, 10.1, 6.7), 5.02 – 4.85 (4 H, m), 4.15 (4 H, q, J 7.1), 2.06 – 1.98 (4 H, m), 1.87 – 1.80 (4 H, m), 1.40 – 1.05 (31 H, m).^{13}C-NMR (101 MHz, $CDCl_3$): 171.97, 139.12, 114.07, 60.85, 57.51, 33.75, 32.09, 29.79, 29.33, 29.24, 29.05, 28.88, 23.87, 14.08.

2) Synthesis of Diethyl 2,2-Di(dec-9-en-1-yl)malonate (M2)

The **M2** was synthesized according to reference [2]. ^{1}H-NMR (400 MHz, DMSO-*d6*: δ ppm 4.12 (q, J = 7.1 Hz, 4H), 2.77 (t, J = 2.6 Hz, 1H), 2.18 (td, J = 6.8, 2.7 Hz, 2H), 1.92 – 1.74 (m, 4H), 1.31 – 1.21 (m, 2H), 1.16 (t, J = 7.1 Hz, 6H).^{13}C-NMR (101 MHz, DMSO-*d6*): δ ppm 171.29, 84.37, 71.90, 61.17, 57.44, 30.59, 25.07, 23.10, 18.31, 14.37, 8.52.

General Synthesis of Barbiturate and 2-Thiobarbiturate

To synthesize different barbiturates and 2-thiobarbiturates by condensation of malonate substrates with urea or thiourea, the general synthesis was modified according to reference [2,3] as follow: to a flask cooled in an ice bath finely grounded urea (10 equivalence) or thiourea (6 equivalence) and potassium tert-butoxide (2.2 equivalence) were added. Dry DMSO was added to dissolve the solids, followed by stirring for 30 minutes till the mixture turned clear. Then the malonate (**M1** or **M2**, 1 equivalence) was added and the mixture was stirred at different temperature for different time, monitored by TLC. After reaction the reaction mixture was diluted with 1 M hydrochloric acid (the pH of the mixture was checked to be 2). The mixture was extracted with ethyl acetate for three times. The combined organic extracts were washed with water, dried with sodium sulfate, filtered, and concentrated under reduced pressure. Further purification was performed by column chromatography on silica or recrystallization in

toluene at 75 °C.

3) Synthesis of 5,5-Di(dec-9-en-1-yl)pyrimidine-2,4,6(1H,3H,5H)-trione (B)

General synthesis was followed. Urea (3.00 g, 50 mmol), potassium tert-butoxide (1.23 g, 11.0 mmol) and diethyl 2,2-di(dec-9-en-1-yl)malonate **M 1** (2.18 g, 5.0 mmol) were mixed in 20 ml DMSO and stirred at 40 °C for 48 hours. Column chromatography on silica with ethyl acetate/*n*-hexane 1:4 was performed. After drying under high vacuum, **B1** as a white solid was obtained. Yield: 1.05 g (52 %). M.p.: 91°C (DSC). ^{1}H-NMR (500 MHz, $CDCl_3$): δ ppm 8.75 (2 H, s), 5.79 (2 H, ddt, *J* 16.9, 10.2, 6.7), 5.02 – 4.89 (4 H, m), 2.07 – 1.90 (8 H, m), 1.42 – 1.10 (24 H, m). ^{13}C-NMR (101 MHz, $CDCl_3$): δ ppm 172.93 , 149.14 , 139.23 , 114.31 , 56.87 , 39.33 , 33.88 , 29.55 , 29.40 , 29.23 , 29.12 , 28.97 , 25.22. ESI-ToF MS: $[M-H]^-$ calculated for $C_{24}H_{40}N_2O_3$, 403.60; found, 403.29.

4) Synthesis of 5,5-Di(dec-9-en-1-yl)-2-thioxodihydropyrimidine-4,6(1H,5H)-dione (TB)

General synthesis was followed. Thiourea (1.14 g, 15.0 mmol), potassium tert-butoxide (617 mg, 5.5 mmol) and **M1** (1.10 g, 2.5 mmol) were mixed in 10 ml DMSO and stirred at room temperature for 24 hours. Column chromatography on silica with diethyl ether/*n*-hexane 1:4 was performed. After drying under high vacuum, **TB** as a slight yellowish high viscous liquid was obtained. Yield: 0.65 g (62 %). ^{1}H-NMR (500 MHz, $CDCl_3$): δ ppm 9.23 (2 H, s), 5.79 (2 H, ddt, *J* 16.9, 10.2, 6.7), 5.02 – 4.89 (4 H, m), 2.06 – 1.88 (8 H, m), 1.43 – 1.11 (24 H, m). ^{13}C-NMR (101 MHz, $CDCl_3$): δ ppm 176.09 , 170.80 , 139.24 , 114.32 , 57.04 , 39.41 , 33.89 , 29.55 , 29.38 , 29.20 , 29.11 , 28.97 , 25.19. ESI-ToF MS: $[M-H]^-$ calculated for $C_{24}H_{40}N_2O_2S$, 419.66; found, 419.26.

5) Synthesis of 5-Ethyl-5-(pent-4-yn-1-yl)pyrimidine-2,4,6(1H,3H,5H)-trione (B2)

The **B2** was synthesized according to reference [2]. ^{1}H-NMR (400 MHz, DMSO-*d6*): δ ppm 11.53 (s, 2H), 2.77 (t, *J* = 2.6 Hz, 1H), 2.12 (td, *J* = 6.9, 2.7 Hz, 2H), 1.91 – 1.78 (m, 4H), 1.30 – 1.21 (m, 2H), 0.74 (t, *J* = 7.4 Hz, 3H). ^{13}C-NMR (101 MHz, DMSO-*d6*): δ ppm 173.29, 150.23, 83.95, 72.12, 55.86, 37.26, 31.78, 24.06, 18.04, 9.51. ESI-ToF MS: $[M-H]^-$ calculated for $C_{11}H_{14}N_2O_3$, 221.24; found, 221.10.

6) Synthesis of 5-Ethyl-5-(pent-4-yn-1-yl)-2-thioxodihydropyrimidine-4,6(1H,5H)-

dione (TB2)

General synthesis was followed. Thiourea (6.45 g, 84.71 mmol), potassium tert-butoxide (3.67 g, 31.06 mmol) and **M2** (3.59 g, 14.12 mmol) were mixed in 15 ml DMSO and stirred at room temperature for 24 hours. Column chromatography on silica with ethyl acetate/chloroform 1:8 was performed then the crude **TB2** was recrystallized in toluene. After drying under high vacuum, **TB2** as a slight yellowish solid was obtained. Yield: 1.85 g (56 %). ^{1}H-NMR (400 MHz, DMSO-*d6*): δ ppm 12.61 (s, 2H), 2.78 (t, *J* = 2.6 Hz, 1H), 2.13 (td, *J* = 6.9, 2.7 Hz, 2H), 1.93 – 1.81 (m, 4H), 1.32 – 1.20 (m, 2H), 0.75 (t, *J* = 7.4 Hz, 3H). ^{13}C-NMR (101 MHz, DMSO-*d6*): δ ppm 179.23, 171.31, 83.91, 72.17, 56.45, 37.20, 31.81, 24.03, 18.02, 9.50. ESI-ToF MS: $[M-H]^-$ calculated for $C_{11}H_{14}N_2O_2S$, 237.31; found, 237.08.

Synthesis of Telechelic Polyisobutylenes

7) Synthesis of Dimethyl-5-(tert-butyl)isophthalate (1)

5-(tert-butyl)isophthalic acid (2.5 g, 11.25 mmol) was dissolved in methanol (100 ml). Then sulfuric acid (1.25 ml, 22.6 mmol) was added and the reaction mixture was heated to reflux for 48 hours. After cooling down to room temperature, the solvent was removed under reduced pressure. The product was purified using column chromatography (chloroform) and allowed to crystallize in the freezer. After drying under vacuum, **1** as a white solid was obtained. Yield: 2.4 g (85 %). ^{1}H NMR (400 MHz, $CDCl_3$): δ 8.50 (t, *J* = 1.6 Hz, 1H), 8.26 (d, *J* = 1.6 Hz, 2H), 3.95 (s, 6H), 1.38 (s, 9H). ^{13}C NMR (100 MHz, $CDCl_3$): δ 166.63, 152.13, 130.91, 130.35, 128.02, 52.27, 34.98, 31.16.

8) Synthesis of 2,2-(5-(Tert-butyl)-1,3-phenylene)bis(propan-2-ol) (2)

A 500 ml flask was heated and flushed with nitrogen thrice. Magnesium chips (4.6 g, 0.19 mol) were dissolved in dry diethyl ether (100 ml) and a small portion of iodomethane (2 ml, 32 mmol) was added. The mixture was allowed to react until a color change was observed. The reaction mixture was held at 0°C, while the rest of iodomethane (12.5 ml, 200 mmol) was added. In a second flask **1** (9.5 g, 38 mmol) was dissolved in dry diethyl ether (150 ml) and was added to the reaction mixture over 2

hours and stirred for 16 hours. The solvent was evaporated und the crude product was recrystallized from hot ethyl acetate at 80°C. After drying under vacuum, **2** as a white solid was obtained. Yield: 8.0 g (84 %). ^{1}H NMR (400 MHz, $CDCl_3$): δ 7.43 (m, 3H), 1.75 (s, 2H), 1.60 (s, 12H), 1.35 (s, 9H). ^{13}C NMR (100 MHz, $CDCl_3$): δ 150.98, 148.63, 119.83, 117.65, 72.88, 35.01, 31.88, 31.50.

9) Synthesis of 1-(Tert-butyl)-3,5-bis(2-methoxypropan-2-yl)benzene (3)

2 (1.9 g, 7.6 mmol) was dissolved in methanol (30 ml). Then sulfuric acid (0.004 ml, 72.3 µmol) was added and the reaction mixture was heated to refluxed for 24 hours. After cooling down to room temperature. The solution was adjusted to pH 7 using sodium bicarbonate, then extracted with hexane (50 ml) twice. The organic phase was then washed with distilled water (50 ml) four times, then dried with sodium sulfate. The solvent was removed under vacuum. After drying under vacuum, **3** as a white solid was obtained. Yield: 1.5 g (80 %). ^{1}H NMR (400 MHz, $CDCl_3$): δ 7.32 (d, *J* = 1.7 Hz, 2H), 7.23 (t, *J* = 1.7 Hz, 1H), 3.07 (s, 6H), 1.54 (s, 12H), 1.33 (s, 9H). ^{13}C NMR (100 MHz, $CDCl_3$): δ 150.76, 145.20, 121.19, 120.35, 77.24, 50.56, 34.84, 31.51, 28.13.

10) Synthesis of Telechelic Dibromopolyisobutylene (PBr)

A 250 ml flask was heated and flushed with nitrogen thrice. A stock solution of DCM (1 ml), N,N-dimethylacetamide (21 µl, 0.23 µmol), di-tert-butyl pyridine (50 µl, 0.23 µmol) and **3** (244 mg, 0.8 mmol) were prepared in a separate flask. Hexane and DCM (45 ml; 60:40) and the stock solution were added to the reaction flask via septum and cooled down to -80°C. Isobutylene (3.4 ml, 35.6 mmol) was condensed at -70°C in a separate flask and added to the reaction flask via septum. Titanium tetrachloride (1.2 ml, 10.9 mmol) was added and the mixture was stirred for 10 min. The reaction was quenched with 1-bromo-3-phenoxypropane (803 µl, 5.1 µmol) and stirred for 3 hours. The solvent was removed under reduced pressure, and the crude polymer was subsequently dissolved in hexane and precipitated into methanol (400 ml). After drying under high vacuum, **PBr** as a colorless viscous liquid was obtained. Yield: 2.7 g (90 %). $M_{n,\,GPC}$ = 2981. ^{1}H NMR (400 MHz, $CDCl_3$): δ 7.26 (m, 4H), 7.17 (s, 3H), 6.82 (m, 4H), 4.08 (t, J = 5.8 Hz, 4H), 3.60 (t, J = 6.5 Hz, 4H), 2.31 (p, J = 6.2 Hz, 4H), 1.83 (s, 6H), 1.80 (s, 6H), 1.42 (m, 85H), 1.10 (m, 256H).

11) Synthesis of Telechelic Diazidopolyisobutylene (PN3)

Telechelic dibromo-PIB **PBr** (M_n was assumed to 3000) (1.15 g, 0.383 mmol) was dissolved in a 80 ml *n*-heptane/DMF mixture (1:1 v/v), then sodium azide (506 mg, 7.7 mmol) was added. The reaction mixture was heated to 90 °C for 8 h. After the reaction mixture was cooled down, the *n*-heptane layer was separated and washed with water (3 x 50 ml). The organic layer was dried over sodium sulfate and the solvent was removed. The crude polymer was redissolved in *n*-hexane and then precipitated into methanol (3 x 400 ml). After drying under high vacuum **PN3** as a yellowish viscous liquid was obtained. Yield: 1.05 g (94 %). $M_{n, GPC}$ = 2818. ^{1}H NMR (400 MHz, $CDCl_3$): δ 7.26 (d, J = 8.8 Hz, 4H), 7.17 (s, 3H), 6.81 (d, J = 8.8 Hz, 4H), 4.03 (t, J = 5.9 Hz, 4H), 3.51 (t, J = 6.7 Hz, 4H), 2.04 (p, J = 6.3 Hz, 4H), 1.83 (s, 6H), 1.80 (s, 6H), 1.44 – 1.36 (m, 85H), 1.18 – 0.95 (m, 256H).

12) Synthesis of Telechelic Dibarbiturate Polyisobutylene (PB)

The synthesis was modified according to reference [4,5]. Telechelic diazido-PIB (**PN3**) (M_n was assumed to 3000) (506.4 mg, 0.169 mmol), **B2** (87.68 mg, 0.371 mmol), and N,N-diisopropylethylamine (174.2 µl, 1.016 mmol) were dissolved in 15 ml dry THF. The mixture was purged with argon for 30 minutes to remove oxygen. Cu(I)iodide (6.56 mg, 0.0338 mmol) was added under counterflow of argon then stirred at 50 °C for 48 hours. The reaction was checked by TLC. After reaction was finished, 30 ml 1 M hydrochloric acid was added and the mixture was extracted with DCM (3 x 30 ml). The combined organic mixture was washed with water (1 x 30 ml) and dried over sodium sulfate. Then DCM was removed under reduced pressure and the raw product was purified by column chromatography on silica with ethyl acetate/hexane (1:1, $R_{f,\ BA}$ = 0.5, $R_{f,\ P1}$ = 0) then chloroform/methanol (1:20, $R_{f,\ P1}$ = 0.5), followed by precipitation into methanol (400 ml). After drying under high vacuum **PB** as a yellowish rubbery solid was obtained. Yield: 265 mg (45 %). $M_{n, GPC}$ = 3165. ^{1}H NMR (400 MHz, $CDCl_3$): 8.88 (s, 4H), 7.29 (s, 2H), 7.26 (d, J = 8.8 Hz, 4H), 7.17 (s, 3H), 6.80 (d, J = 8.8 Hz, 4H), 4.53 (t, J = 6.8 Hz, 4H), 3.95 (t, J = 5.7 Hz, 4H), 2.68 (t, J = 7.5 Hz, 4H), 2.35 (p, J = 6.4 Hz, 4H), 2.03 (dq, 4H), 1.83 (s, 6H), 1.80 (s, 6H), 1.70 – 1.53 (m, 8H), 1.43

– 1.30 (m, 85H), 1.14 – 1.00 (m, 256H), 0.88 (t, J = 7.4 Hz, 6H).

13) Synthesis of Telechelic Dithiobarbiturate Polyisobutylene (PTB)

The synthesis was modified according to reference [4,5]. Telechelic diazido-PIB (**PN3**) (M_n was assumed to 3000) (434.6 mg, 0.145 mmol), **TB2** (78.64 mg, 0.33 mmol), and N,N-diisopropylethylamine (257.67 µl, 1.5 mmol) were dissolved in 15 ml dry THF. The mixture was purged with argon for 30 minutes to remove oxygen. Cu(I)iodide (5.83 mg, 0.033 mmol) was added under counterflow of argon then stirred at 50 °C for 48 hours. The reaction was checked by TLC. After reaction was finished, 30 ml 1 M hydrochloric acid was added and the mixture was extracted with DCM (3 x 30 ml). The combined organic mixture was washed with water (1 x 30 ml) and dried over sodium sulfate. Then DCM was removed under reduced pressure and the raw product was purified by column chromatography on silica with chloroform/hexane (1:4, $R_{f,\ PTB}$ = 0, $R_{f,\ TB2}$ = 0, $R_{f,\ PN3}$ = 1), then chloroform ($R_{f,\ PTB}$ = 0, $R_{f,\ TBA}$ = 0.25), and final methanol/chloroform (1:20, $R_{f,\ PTB}$ = 0.5) followed by precipitation into methanol (3 x 400 ml). After drying under high vacuum **PTB** as a brown rubbery solid was obtained. Yield: 230 mg (49 %). $M_{n,\ GPC}$ = 3165. ^1H NMR (400 MHz, $CDCl^3$) δ 9.24 (s, 4H), 7.27 (d, J = 8.5 Hz, 6H), 7.17 (s, 3H), 6.80 (d, J = 8.7 Hz, 4H), 4.54 (t, J = 6.7 Hz, 4H), 3.95 (t, J = 5.6 Hz, 4H), 2.74 – 2.60 (m, 4H), 2.36 (p, J = 6.4 Hz, 4H), 2.04 (dq, J = 16.0, 8.4 Hz, 4H), 1.83 (s, 6H), 1.80 (s, 6H), 1.61 (s, 8H), 1.47 – 1.29 (m, 1036H), 1.15 – 0.99 (m, 2345H), 0.89 (t, 25H).

2. Nonlinear Fitting of NH Chemical Shift for Association Constants

For determination of the dimerization constant of the barbiturate **B** and thiobarbiturate **TB**, the stock solutions in $CDCl_3$ were prepared and the exact concentration was calculated. The NMR samples were prepared by diluting the exact amount of the stock solutions with $CDCl_3$ to reach the desired concentration, varying from 0.005 to 0.1 M. Then the N-H chemical shifts were recorded and plotted *vs* concentration as shown in the main text in Figure 1c). The association constant was calculated using the following equation (1):

$$\delta = \delta_{NH,free} + \frac{\delta_{NH,max} - \delta_{NH,free}}{[B]_0}\left([B]_0 + \frac{1}{4K_{assn.}} - \sqrt{([B]_0 + \frac{1}{4K_{assn.}})^2 - [B]_0^2}\right) \quad (1)$$

Where

δ is the experimental N-H chemical shift;

$\delta_{NH,free}$ is the N-H chemical shift for the species free from hydrogen bonds (the N-H chemical shift from the sample with 0.005 M was used here);

$\delta_{NH,max}$ is the N-H chemical shift for the fully hydrogen bonded species;

$[B]_0$ is the concentration of the NMR sample;

$K_{assn.}$ is the association constant.

3. Calculation of Coalescence Constant

Using the equation (2), the coalescence rate constant at the $T_{Coalescence}$ = 0 °C can be calculated.

$$k_c = \frac{\pi\Delta\upsilon}{\sqrt{2}} \approx 2.22\sqrt{\Delta\upsilon^2 + J_{AB}^2} \quad (2)$$

Where

$\Delta\upsilon$ is the difference of frequency of the two isolated peaks;

J_{AB} is the *J*-coupling of the two isolated peaks

If we apply the coalescence rate constant to Eyring equation (3), the free enthalpy of coalescence can be calculated.

$$k_c = \chi\frac{k_B T}{h} e^{\Delta G_c^{\neq}/RT} \quad (3)$$

Where

k_B is the Boltzmann constant;

χ is the transmission coefficient, assumed equal to 1;

h is the Planck constant:

$\Delta G_c^{\neq}$ is the Gibbs free energy of activation.

4. Space Filling Model of B in Langmuir Film

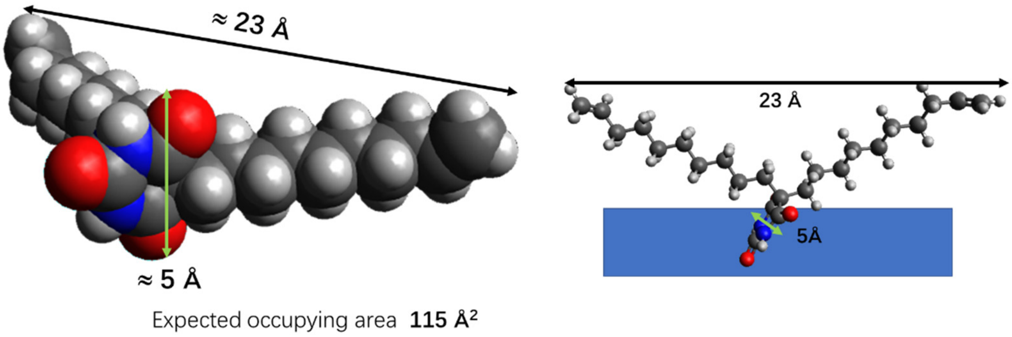

Figure S2 The space filling model of **B** and the hypothesized arrangement on the water subphase. For the molecule **TB**, the model is identical except a larger C2=S thus is not shown here.

5. Summary of Characterization of Model Polymers

Table S1 Molecular weight and PDI from NMR, GPC, and MALDI-ToF MS; glass transition temperature; and melt viscosity at 80 °C of polymer precursor **PBr** and model polymer **PB** and **PTB**.

	$M_{n\ NMR}$	$M_{n\ GPC}$	$M_{n\ MALDI}$	PDI_{GPC}	$T_{g\ DSC}$ (°C)	Viscosity $_{80°C}$ (Pa·s)
**PBr**	3512	2981	-	1.32	-58.8	6.43
**PB**	3741	3165	2800	1.43	-61.3	3.03×10^7
**PTB**	3971	3244	3091	1.78	-59.6	1.63×10^8

6. NMR Spectra of Model Compounds and Polymers

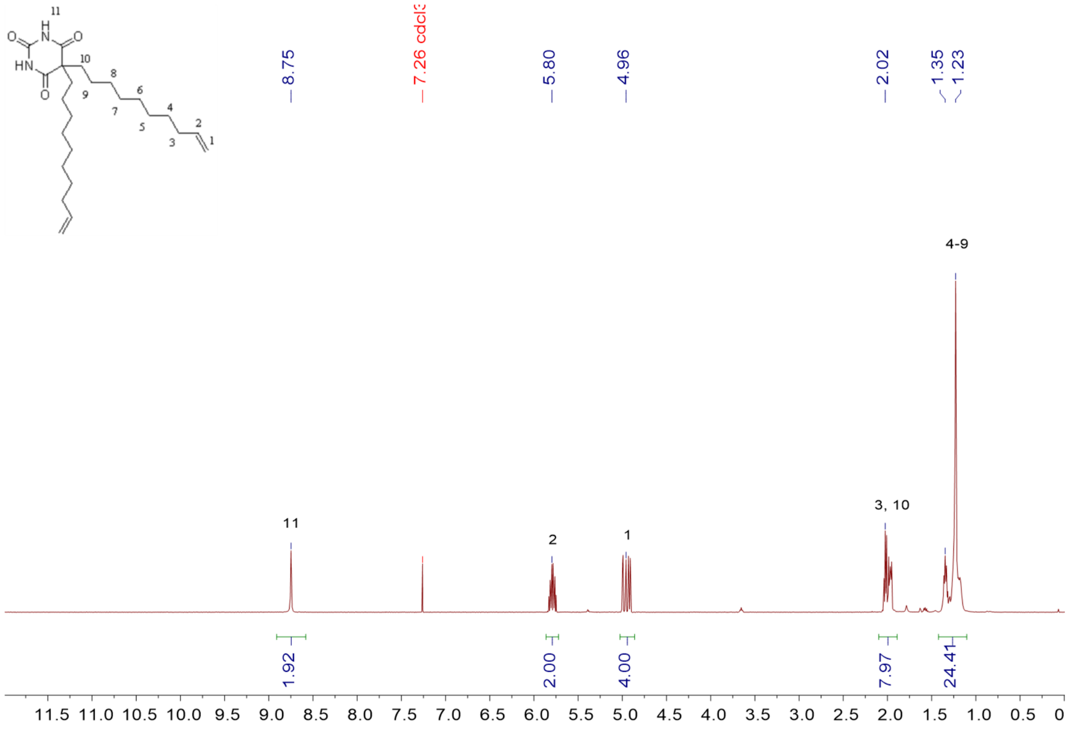

Figure S3 ^{1}H NMR spectrum of **B**.

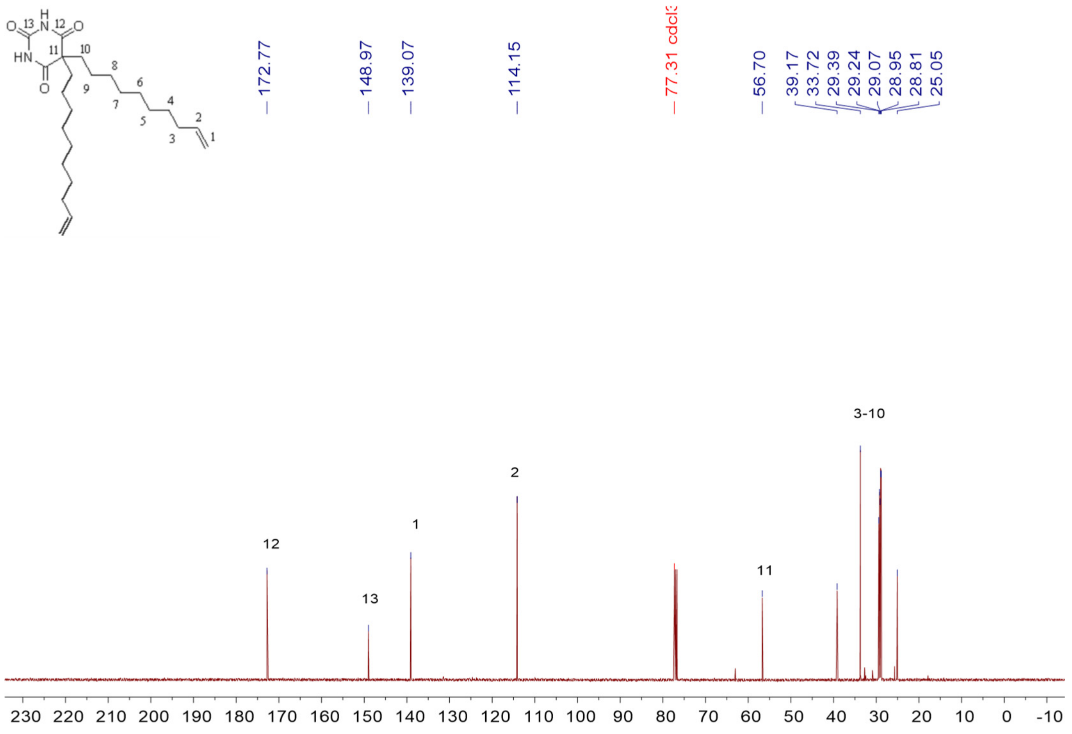

Figure S4 ^{13}C NMR spectrum of **B**.

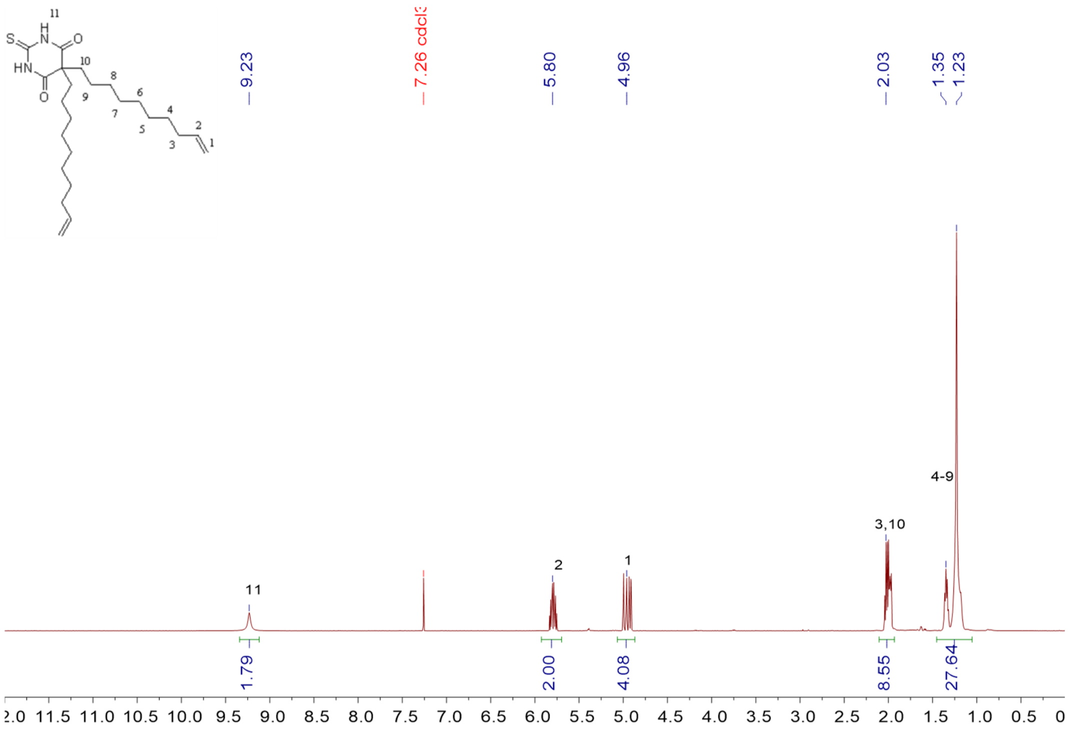

Figure S5 ^{1}H NMR spectrum of **TB**.

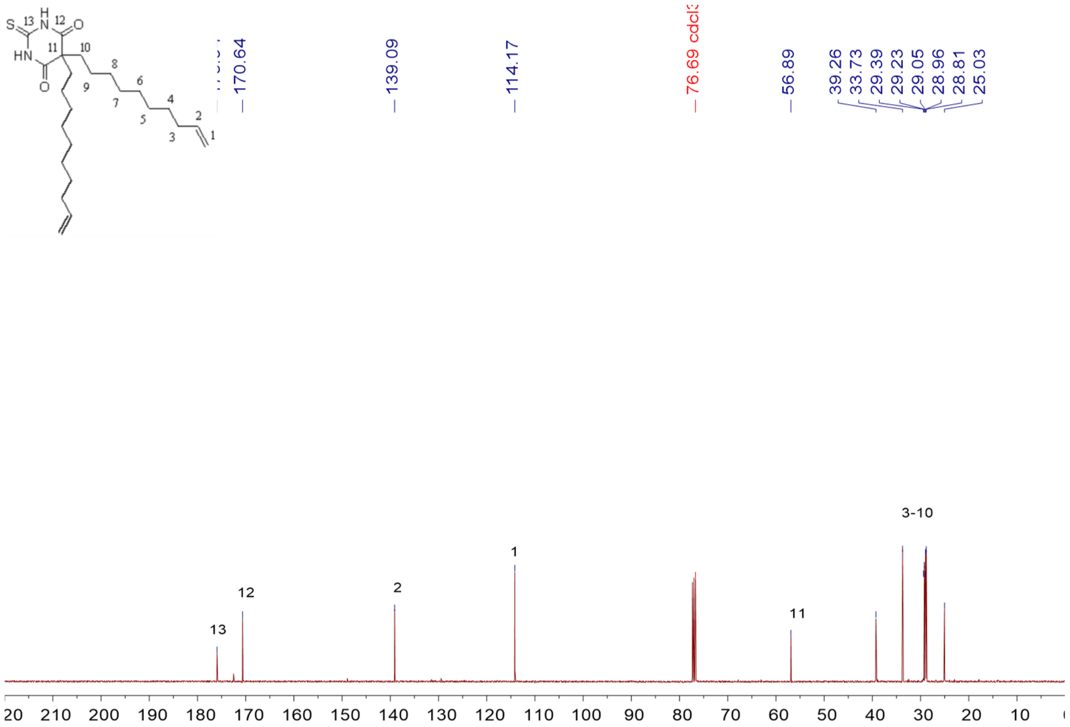

Figure S6 ^{13}C NMR spectrum of **TB**.

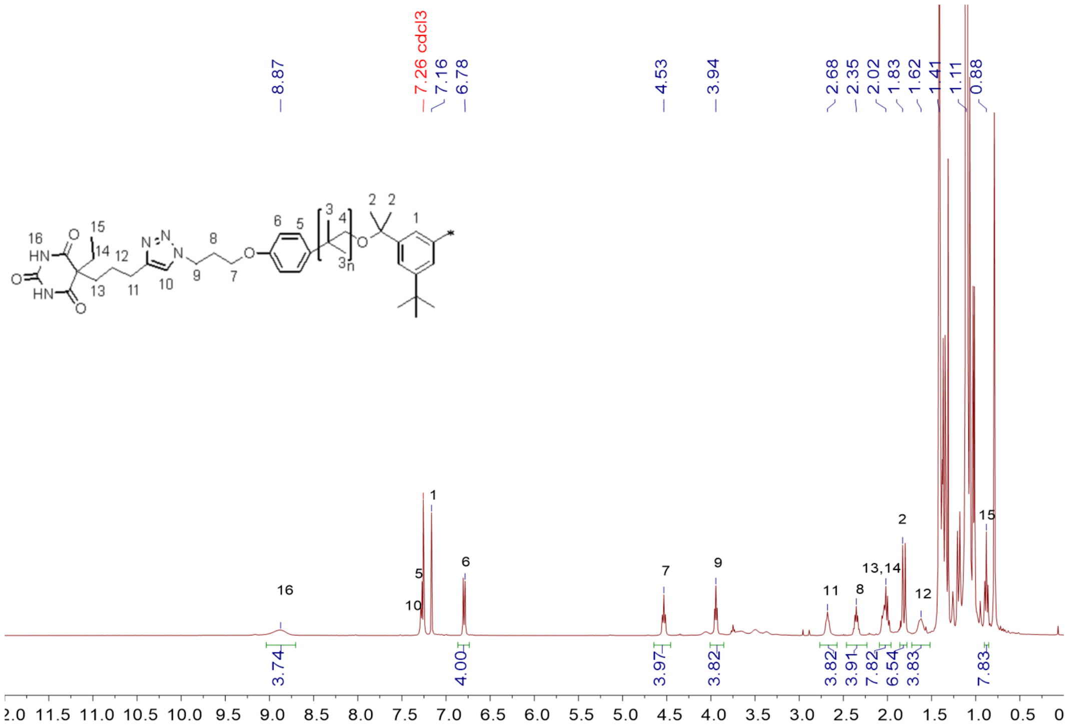

Figure S7 ^{1}H NMR spectrum of **PB**.

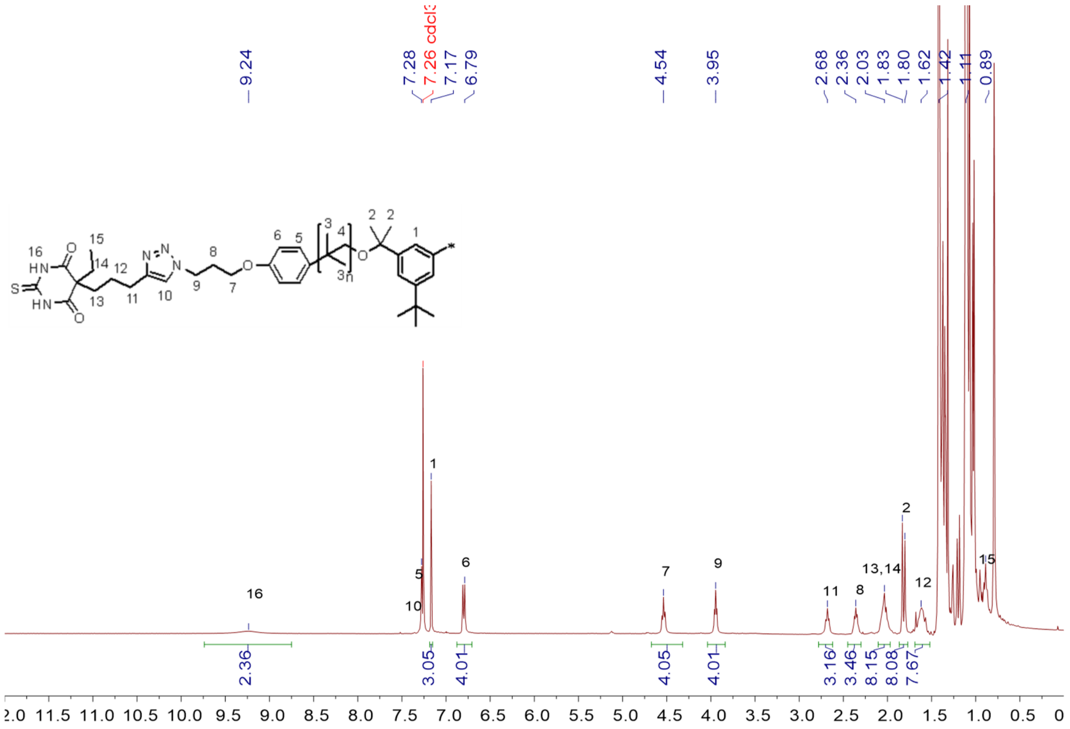

Figure S8 ^{1}H NMR spectrum of **PTB**.

7. ESI-ToF MS Spectra of Model Compounds

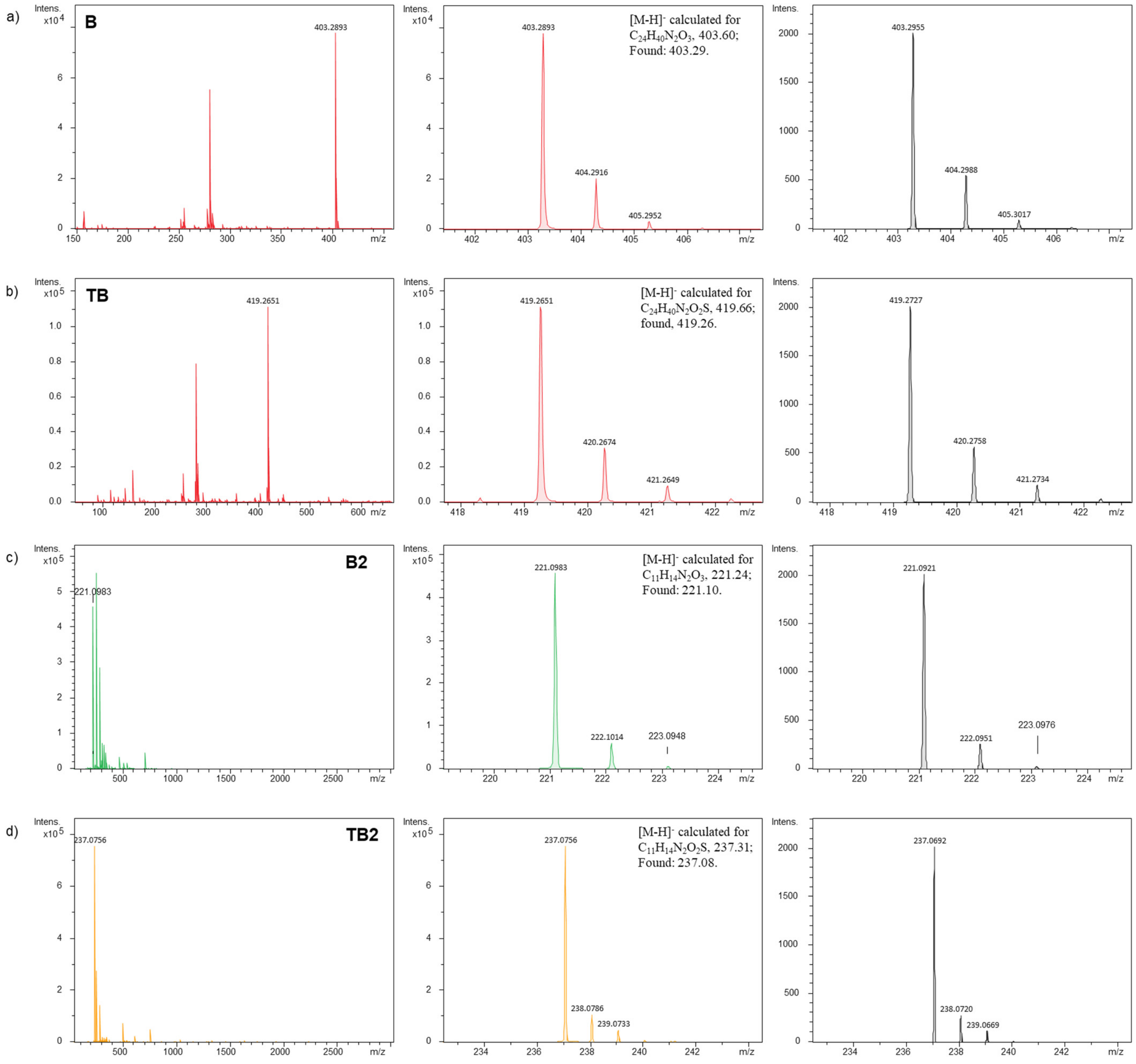

Figure S9 Left: the full experimental ESI-ToF MS spectra; middle: the compound spectra; and right : the simulated isotope pattern of a) **B**, b) **TB**, c) **B2**, and d) **TB2**.

8. MALDI-ToF MS Spectra of Model Polymers

1,8-dihydroxy-9,10-dihydroanthracen-9-one (Dithranol, 20 mg·mL^{-1} in THF) was used as matrix and sodium iodide (NaI, 20 mg·mL^{-1} in THF) was used as salts for ionizing polymers functionalized with hydrogen-bonding moieties (model polymer **PB** or **PTB**, 20 mg·mL^{-1} in THF) while applying a volume ratio of 100:20:1 in reflection mode.

8.1. Spectra of PB

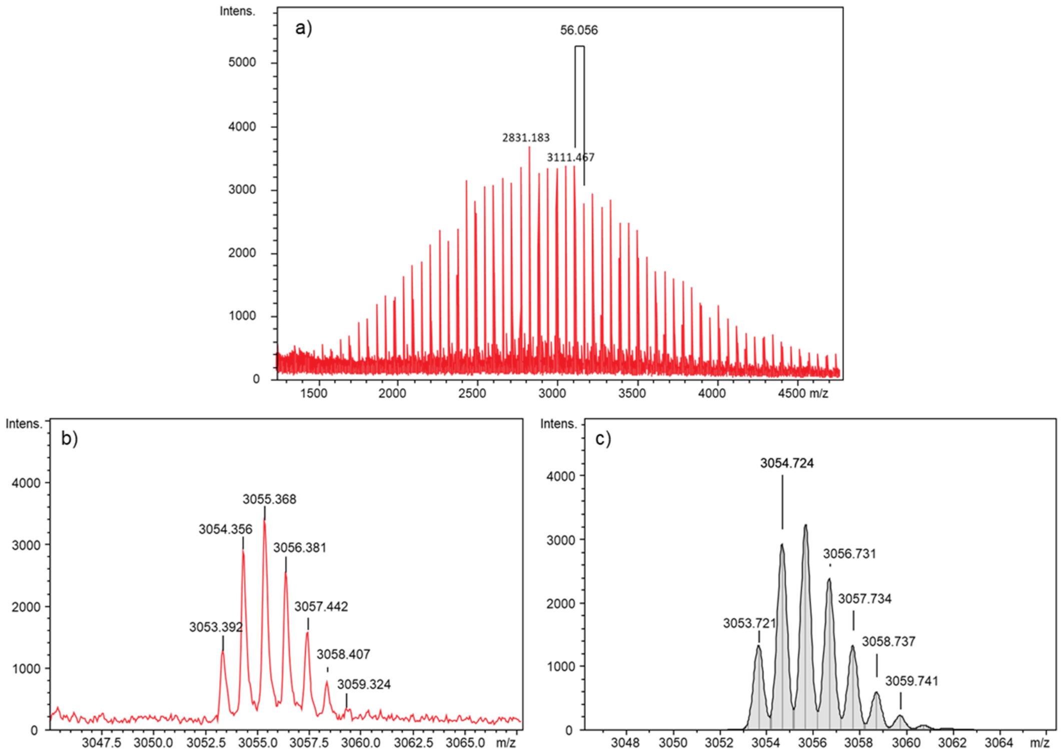

Figure S10 a) MALDI-ToF spectra of **PB**; b) the zoom-in of the peak at 3055.37; and c) the simulated isotope pattern for [C36H48N5O6(C4H8)35C20H24N5O4]+[Na2+H]$^{+}$.

8.2. Spectra of PTB

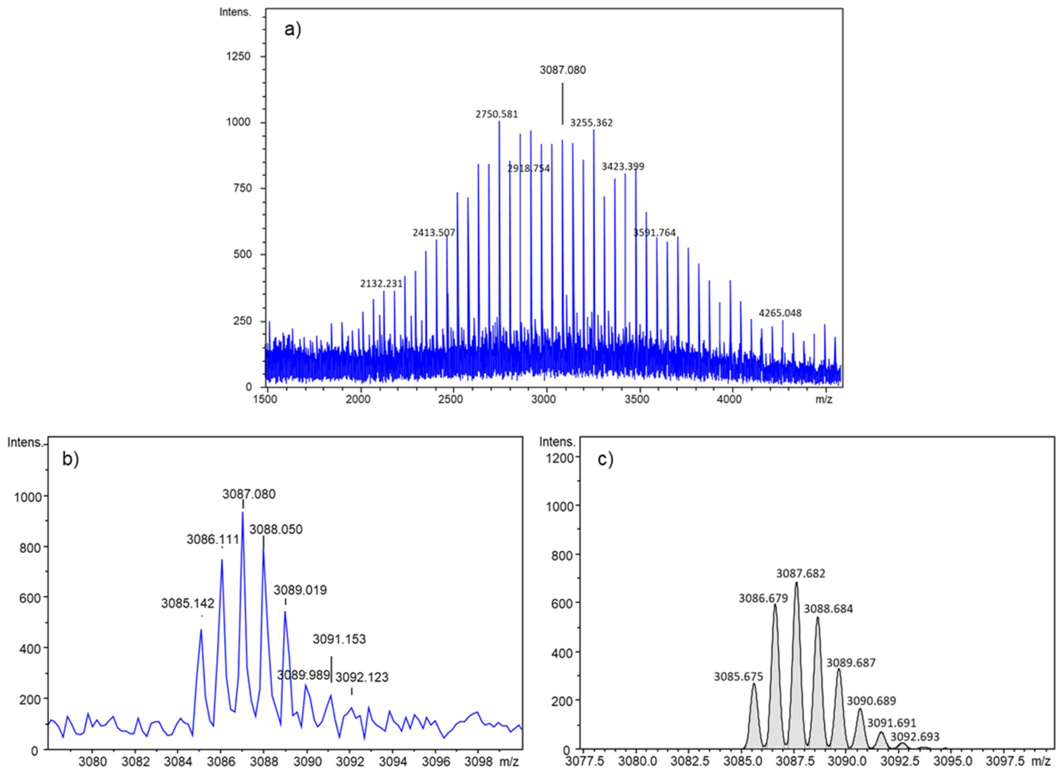

Figure S11 a) MALDI-ToF spectra of **PTB**; b) the zoom-in of the peak at 3087.08; and c) the simulated isotope pattern for [C36H48N5O5S(C4H8)35C20H24N5O3S]+[Na2+H]$^{+}$.

9. DSC Curves of Model Polymers

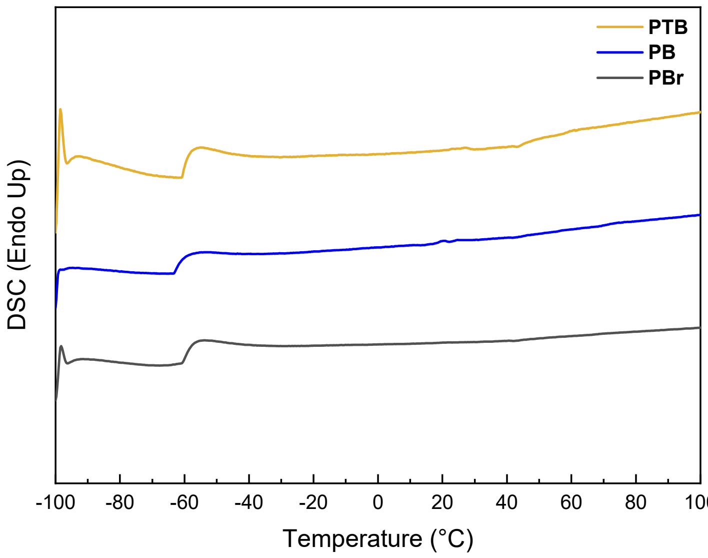

Figure S12 DSC curves of telechelic dibromo PIB **PBr**, model polymer **PB** and **PTB** (curves are vertically shifted).

10. Loss Tangent vs Frequency Curve of Model Polymers

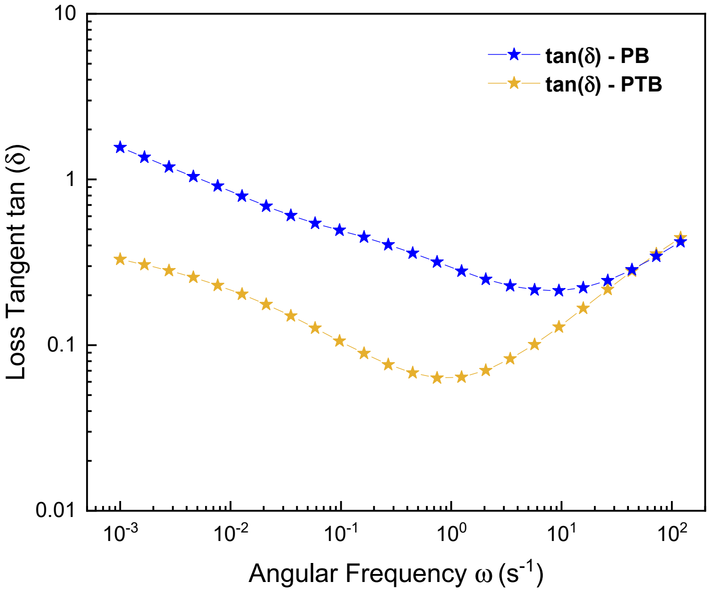

Figure S13 Loss tangent *vs* frequency curve of model polymer **PB** and **PTB**.

11. Zero-shear Viscosity vs Temperature Curve of Model Polymers

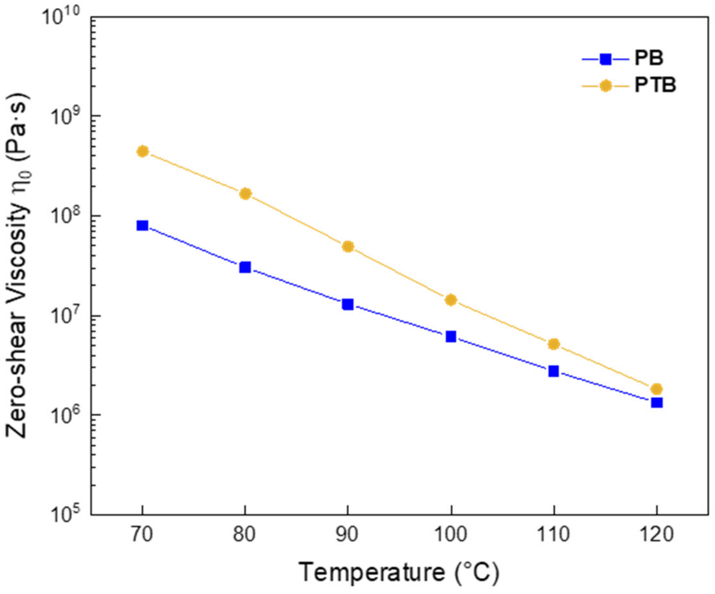

Figure S14 Zero-shear viscosity *vs* temperature curve of model polymer **PB** and **PTB**.

12. Reference

1. Kotha, S.D., Ashoke Chandra. Design and synthesis of spiro-heterocycles by ring-closing metathesis. *Indian Journal of Chemistry* **2008**, *47 B*, 1120-1134.
2. Srivastava, P.C.; Callahan, A.P.; Cunningham, E.B.; Knapp, F.F. Potential cerebral perfusion agents: synthesis and evaluation of a radioiodinated vinylalkylbarbituric acid analog. *Journal of Medicinal Chemistry* **1983**, *26*, 742-746, doi:10.1021/jm00359a020.
3. Bouhlel, A.; Curti, C.; Vanelle, P. New methodology for the synthesis of thiobarbiturates mediated by manganese(III) acetate. *Molecules* **2012**, *17*, 4313-4325, doi:10.3390/molecules17044313.
4. Schulz, M.; Glatte, D.; Meister, A.; Scholtysek, P.; Kerth, A.; Blume, A.; Bacia, K.; Binder, W.H. Hybrid lipid/polymer giant unilamellar vesicles: effects of incorporated biocompatible PIB–PEO block copolymers on vesicle properties. *Soft Matter* **2011**, *7*, 8100-8110, doi:10.1039/C1SM05725A.
5. Rupp, H.; Döhler, D.; Hilgeroth, P.; Mahmood, N.; Beiner, M.; Binder, W.H. 3D Printing of Supramolecular Polymers: Impact of Nanoparticles and Phase Separation on Printability. *Macromolecular Rapid Communications* **2019**, *40*, 1900467, doi:10.1002/marc.201900467.
